# RNF40 regulates gene expression in an epigenetic context-dependent manner

**DOI:** 10.1186/s13059-017-1159-5

**Published:** 2017-02-16

**Authors:** Wanhua Xie, Sankari Nagarajan, Simon J. Baumgart, Robyn Laura Kosinsky, Zeynab Najafova, Vijayalakshmi Kari, Magali Hennion, Daniela Indenbirken, Stefan Bonn, Adam Grundhoff, Florian Wegwitz, Ahmed Mansouri, Steven A. Johnsen

**Affiliations:** 1 Department of General, Visceral and Pediatric Surgery, Göttingen Center for Molecular Biosciences, University Medical Center Göttingen, Justus-von-Liebig-Weg 11, 37077 Göttingen, Germany; 2 Research Group for Computational Systems Biology, German Center for Neurodegenerative Diseases (DZNE), Griesebachstraße 5, 37077 Göttingen, Germany; 3 Heinrich Pette Institute, Leibniz Institute for Experimental Virology, 20251 Hamburg, Germany; 4 Department of Molecular Cell Biology, Max-Planck Institute for Biophysical Chemistry, Am Fassberg, 37077 Göttingen, Germany; 5 Department of Clinical Neurophysiology, University of Göttingen, Robert-Koch-Strasse 40, 37075 Göttingen, Germany

**Keywords:** RNF40, H2Bub1, Broad H3K4me3, *Ezh2*, Epigenetics, Enhancer, FOXL2

## Abstract

**Background:**

Monoubiquitination of H2B (H2Bub1) is a largely enigmatic histone modification that has been linked to transcriptional elongation. Because of this association, it has been commonly assumed that H2Bub1 is an exclusively positively acting histone modification and that increased H2Bub1 occupancy correlates with increased gene expression. In contrast, depletion of the H2B ubiquitin ligases RNF20 or RNF40 alters the expression of only a subset of genes.

**Results:**

Using conditional *Rnf40* knockout mouse embryo fibroblasts, we show that genes occupied by low to moderate amounts of H2Bub1 are selectively regulated in response to *Rnf40* deletion, whereas genes marked by high levels of H2Bub1 are mostly unaffected by *Rnf40* loss. Furthermore, we find that decreased expression of RNF40-dependent genes is highly associated with widespread narrowing of H3K4me3 peaks. H2Bub1 promotes the broadening of H3K4me3 to increase transcriptional elongation, which together lead to increased tissue-specific gene transcription. Notably, genes upregulated following *Rnf40* deletion, including *Foxl2*, are enriched for H3K27me3, which is decreased following *Rnf40* deletion due to decreased expression of the *Ezh2* gene. As a consequence, increased expression of some RNF40-“suppressed” genes is associated with enhancer activation via FOXL2.

**Conclusion:**

Together these findings reveal the complexity and context-dependency whereby one histone modification can have divergent effects on gene transcription. Furthermore, we show that these effects are dependent upon the activity of other epigenetic regulatory proteins and histone modifications.

**Electronic supplementary material:**

The online version of this article (doi:10.1186/s13059-017-1159-5) contains supplementary material, which is available to authorized users.

## Background

Over the past decade, significant advances have been made in the understanding of the functional role of post-translational modifications of the four core histones. The monoubiquitination of histone H2B on lysine 123 in yeast or lysine 120 in mammals is catalyzed by Bre1 in yeast and the obligate RNF20/RNF40 heterodimeric complex in mammals [1–4]. While its precise mechanisms of action remain largely unknown, H2Bub1 has been suggested to play multiple roles in chromatin-associated molecular processes including gene transcription [5, 6], DNA damage response [7], DNA replication [8], and messenger RNA (mRNA) processing [9, 10].

A significant amount of accumulating data suggests that high H2Bub1 levels are coupled with gene activation and the opening of the chromatin structure [11, 12]. However, while H2Bub1 occupancy is generally correlated with gene expression levels, small interfering RNA (siRNA)-mediated knockdown of either RNF20 or RNF40 affected only a subset of H2Bub1-occupied genes in human cells [6, 11]. While H2Bub1 occupancy is tightly coupled with transcriptional elongation rates [13], knockdown of RNF20 generally did not affect RNA Polymerase II elongation rate in HCT116 cells [14].

Stimulus-induced genes, which presumably require rapid changes in chromatin structure to become active, appear to particularly require H2Bub1 to facilitate recruitment of the FACT histone chaperone complex and induce dynamic changes in chromatin structure [15–17]. One study proposed that the depletion of H2Bub1 had a stronger impact on rapidly transcribed genes, while having fewer effects on highly transcribed genes [13]. In contrast to its apparent positive role in transcription, H2Bub1 was also reported to repress transcription of a subset of genes by blocking the recruitment of the transcriptional elongation factor TFIIS to chromatin [18]. Notably, the vast majority of genes whose expression increased in RNF20-depleted human cells did not display significant levels of H2Bub1, thereby suggesting that “repressive” functions of H2Bub1 likely occur via indirect mechanisms [11].

H2Bub1 was shown to promote the activity of the SET1/COMPASS methyltransferase complex to directly stimulate H3K4 trimethylation [19, 20]. Thus, the trans-histone crosstalk between H2Bub1 and H3K4me3 may be important for the regulation of *Trithorax*- and *Polycomb*-regulated genes such as the *Hox* genes [4]. However, until now genome-wide data investigating this trans-histone crosstalk and its functions on gene transcription are lacking, therefore leaving the importance of this crosstalk unclear. Interestingly, recent studies showed that a loss of H2Bub1 impaired stem-cell differentiation with decreased induction of lineage-specific genes [21–23]. In other recent studies, a class of cell identity-related genes which display broad H3K4me3 domains near their transcriptional start sites (TSSs) was identified [24–27]. However, the mechanisms governing broad H3K4me3 domains and its transcriptional functions as well as any potential connection to H2Bub1 remain unknown. In addition, while increased breadth of H3K4me3 peaks is highly correlated with positive transcription elongation factors [24], the regulatory relationship between H3K4me3 domain width and the elongation machinery is unknown.

In this study, we performed genome-wide studies for H2Bub1, H3K4me3, H3K27me3, and H3K27ac occupancy in inducible *Rnf40* knockout mouse embryo fibroblasts (MEF) and observed that low and moderate levels of H2Bub1 are particularly associated with RNF40-dependent gene expression changes. Interestingly, RNF40-mediated H2B monoubiquitination is required for the formation and maintenance of broad H3K4me3 domains. Consistently, RNF40-dependent genes show broader H3K4me3 peaks near the TSS, which are associated with an increased elongation rate. In addition, the CDK9-RNF20/RNF40 axis-driven H2B monoubiquitination promotes the broadening of H3K4me3 peaks to facilitate tissue-specific gene transcription. While downregulation of gene expression in response to *Rnf40* deletion appears to be largely mediated by H3K4me3-dependent histone crosstalk, the upregulation of many genes, including *Foxl2*, was dependent upon a loss of *Ezh2* transcription and decreased H3K27me3 near TSSs. Many other upregulated genes not displaying significant H3K27me3 prior to *Rnf40* loss were found to be associated with the activation of FOXL2-bound enhancers and dependent upon *Foxl2* expression. Together these findings uncover a previously unknown function of RNF40-mediated H2B monoubiquitination in promoting the broadening of H3K4me3 peaks to increase the transcriptional elongation rates of tissue-specific genes, as well as in the indirect repression of gene transcription via the maintenance/activation of PRC2 and indirect repression of *Foxl2* transcription and provide further insight into the context-dependent intricacies of epigenetic regulation.

## Results

### RNF40-regulated genes display low and moderate H2Bub1 occupancy

**Fig. 1:**
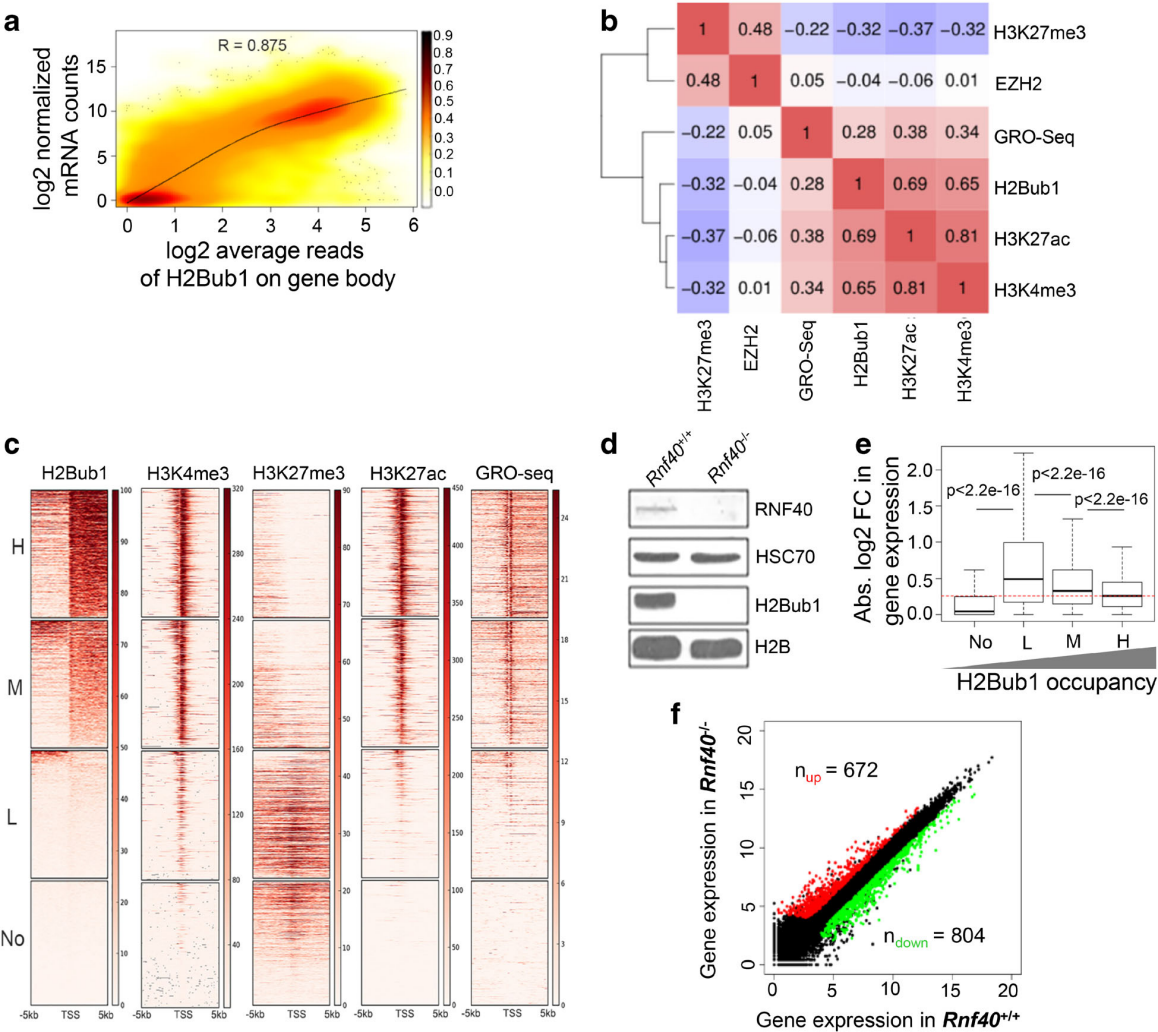
Loss of *Rnf40* alters gene expression on genes displaying low or moderate levels of H2Bub1. **a** *SmoothScatter plot analysis* displays the relationship between H2Bub1 occupancy on the gene body compared to the mRNA levels of the corresponding gene. Gene density is indicated on the *right*. The correlation coefficient is calculated with the “Pearson” method. **b** *Correlation plot* shows the *heatmap* with the Pearson correlation coefficients for H2Bub1, H3K4me3, H3K27me3, H3K27ac, GRO-Seq, and EZH2 on the region 1 kb downstream of the TSS of all genes. **c** The *heatmaps* show H2Bub1, H3K4me3, H3K27me3, H3K27ac, and GRO-seq levels surrounding the TSS (±5 kb) of all genes in wild-type (*Rnf40* ^+/+^) MEFs. TSSs are sorted according to H2Bub1 level in descending order. The color key is shown on the *right*. According to H2Bub1 occupancy, genes are grouped into high (“H”) displaying genes ranked in the upper 25th percentile, moderate (“M”) in the 50th to 75th percentile of ranked genes, low (“L”) in the 25th to 50th of ranked genes, and the lowest 25th percentile (“No”) of ranked genes. **d** *Western blot analysis* for RNF40 and H2Bub1 levels in *Rnf40* ^+/+^ and *Rnf40*-null (*Rnf40* ^–/–^) MEFs. HSC70 and H2B are shown as loading controls. To induce *Rnf40* knockout, cells were treated with 250 nM of (Z)-4-Hydroxytamoxifen (4-OHT) for five days. **e** *Boxplot* compares the absolute value of log_2_-fold changes in gene expression for the defined groups. *p* values were calculated by unpaired Wilcoxon-Mann-Whitney-Test. **f** *Correlation plot* compares the log_2_-normalized counts of gene expression between *Rnf40* ^+/+^ and *Rnf40* ^–/–^ MEFs. Differentially regulated genes following *Rnf40* deletion were defined as upregulated genes (*red*, baseMean > 15, *p* value < 0.05, log_2_-fold change > 1) and downregulated genes (*green*, baseMean > 15, *p* value < 0.05, log_2_-fold change < –1)

To examine the association between H2Bub1 and gene transcription in detail, we performed chromatin immunoprecipitation analyses coupled with high throughput sequencing (ChIP-seq) of H2Bub1 and correlated these with mRNA levels via mRNA sequencing (RNA-seq) in MEFs. In agreement with the previous findings in human cells [12, 28], H2Bub1 is selectively localized on gene bodies where it is enriched near the 5’ region and gradually decreases toward the 3’ end (Additional file 1: Figure S1A). In addition, H2Bub1 occupancy is highly correlated with gene expression and the active histone marks H3K4me3 and H3K27ac, but negatively correlated with the occupancy of the repressive mark H3K27me3 and its methyltransferase EZH2 (Fig. 1a–c, Additional file 1: Figure S1B).

To investigate the role of RNF40-directed H2B monoubiquitination in gene expression, we developed a conditional knockout mouse in which exons 3 and 4 of the mouse *Rnf40* gene were flanked by LoxP sites (Additional file 1: Figure S1C). This mouse was subsequently crossed to a transgenic line expressing a ubiquitously expressed tamoxifen-inducible Cre recombinase (Rosa26-Cre^ERT2^) and MEF were then obtained from homozygous *Rnf40* ^loxP/loxP^ embryos containing the Cre^ERT2^ transgene [29]. Deletion of *Rnf40* and the resulting loss of H2Bub1 were effectively achieved by treating MEFs with 250nM 4-hydroxytamoxifen (4-OHT) (Fig. 1d). After categorizing genes globally into four clusters based on their degree of H2Bub1 occupancy from high to non-enriched (Fig. 1c), we further observed that genes displaying either undetectable or abundant levels of H2Bub1 were largely unaffected in their expression levels. In contrast, genes displaying low (L) or moderate (M) H2Bub1 occupancy were highly regulated in *Rnf40*-deficient MEFs (Fig. 1c and e). In agreement with earlier work [6], loss of H2Bub1 resulted in changes in expression of a select subset of genes (Fig. 1f).

We next examined the effects of loss of H2Bub1 on the occupancy of H3K4me3, H3K27ac, and H3K27me3 near the TSS. SmoothScatter analysis showed that active marks (H3K4me3 and H3K27ac) were most strongly decreased on genes displaying high levels of H2Bub1 and slightly increased on non-/low-H2Bub1 marked genes (Additional file 1: Figure S1D). Consistent with the dynamic pattern of gene regulation (Fig. 1e), the active and repressive histone marks near the TSS of “L” and “M” gene clusters were significantly altered in *Rnf40*-deficient MEFs (Additional file 1: Figure S1E). Notably, genes in the highly regulated clusters (L and M) displayed a high degree of occupancy of both active and repressive marks (Fig. 1c). We hypothesized that the significant differential expression of the “L” and “M” genes may be associated with changes in the active and repressive histone modifications in *Rnf40*-deficient MEFs.

### Transcriptional dependency on H2Bub1 is linked to widespread narrowing of H3K4me3 peaks

**Fig. 2:**
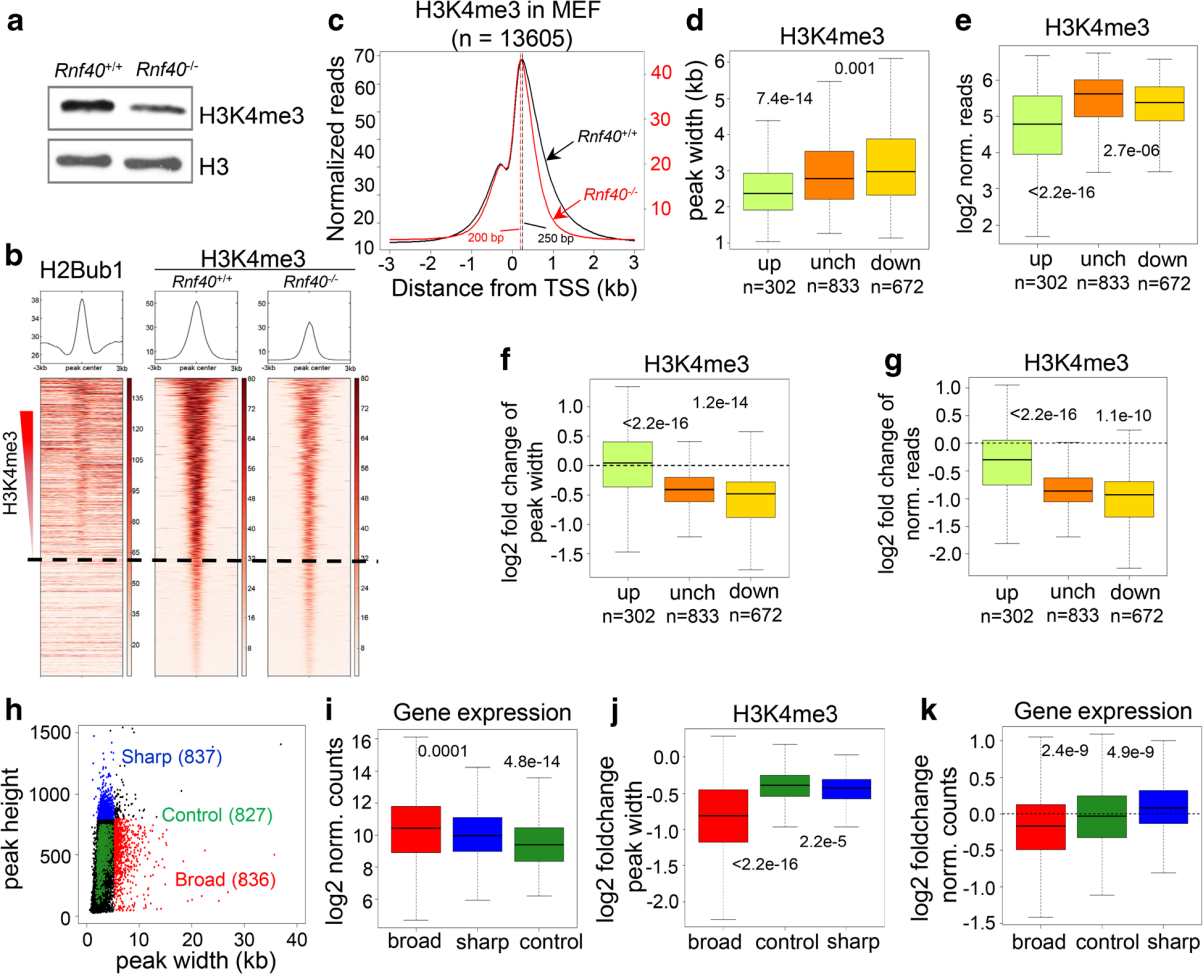
H2Bub1 controls H3K4me3 spreading into gene body to regulate transcription. **a** *Western blot* shows H3K4me3 levels after *Rnf40* deletion. *Western blot* was performed using the same samples shown in Fig. 1d. **b** *Aggregate profiles* (*top*) and *heatmaps* (*bottom*) show the occupancy of H2Bub1 and H3K4me3 surrounding the peak center of H3K4me3 occupied regions (±3 kb). All regions were sorted according to H3K4me3 occupancy from high to low. Color key for each *heatmap* is shown on the *right*. The *dotted line* classified H2Bub1 enriched (*top*) and unenriched (*bottom*) regions. **c** *Aggregate profiles* with different scales compare the average density of H3K4me3 surrounding the TSS of all H3K4me3-enriched genes in *Rnf40* ^+/+^ (*black*) and *Rnf40* ^–/–^ MEFs (*red*). Regions significantly enriched for H3K4me3 on were identified using MACS (cutoff *p* value < 0.00001). The *dotted lines* denote the summits of H3K4me3 peaks in *Rnf40* ^+/+^ (*black*) or *Rnf40* ^–/–^ (*red*) MEFs. **d** *Boxplot* comparing the width of H3K4me3 peaks in *Rnf40* ^+/+^ MEF for H3K4me3 occupied genes upregulated (up), unchanged (unch), or downregulated (down) following *Rnf40* deletion. Upregulated and downregulated genes were defined as in Fig. 1f. Unchanged genes were selected BaseMean > 15, *p* value > 0.8, –0.2 < log_2_-fold change < 0.2. *p* values for differences between the three groups were calculated using unpaired Wilcoxon-Mann-Whitney-Test. **e** *Boxplot* comparing the log_2_-normalized reads of H3K4me3 at promoters (±2 kb) of upregulated, unchanged, and downregulated genes. **f**, **g** *Boxplots* comparing the log_2_-fold change in H3K4me3 peak width (**f**) and height (**g**) in the three cluster genes following *Rnf40* deletion. Log_2_ fold change values in H3K4me3 width or the normalized H3K4me3 reads were calculated as log_2_ (levels in *Rnf40* ^–/–^) – log_2_ (levels in *Rnf40* ^+/+^). *p* values between the three groups were calculated using the unpaired Wilcoxon-Mann-Whitney-Test. **h** *Dotplot analysis* displays the width and height of H3K4me3 peaks near TSS in MEFs. Genes with top 5% broadest (*red*), sharpest (*blue*), and a similar number of random control (*forest green*) H3K4me3-enriched regions were selected based on the described method [25]. **i**–**k** *Boxplots* compare log_2_ normalized gene expression counts (**i**), log_2_-fold change in H3K4me3 peak width (**j**), and log_2_-fold change in gene expression counts (**k**) in each of the given three groups identified in (**h**)

Consistent with an intimate crosstalk between H2Bub1 and H3K4me3, genome-wide H3K4me3 closely paralleled H2Bub1 (Fig. 2b). Moreover, RNF40 deficiency resulted in a genome-wide decrease of H3K4me3 occupancy on H2Bub1-enriched regions, but not on regions lacking H2Bub1 enrichment (Fig. 2a and b; Additional file 1: Figure S2A). In addition, aggregate plot analysis of H3K4me3 average signals revealed that the decrease in H3K4me3 (red line) occupancy was most pronounced at the 3’ side of the TSS-associated H3K4me3 peak coinciding with H2Bub1 occupancy (Additional file 1: Figure S2B). We further investigated the effect of H2Bub1 loss on H3K4me3 occupancy separately at CpG-enriched or -unenriched promoters where H3K4me3 density showed a significant decrease irrespective of CpG composition following *Rnf40* deletion (Additional file 1: Figure S2C). Thus, we conclude that H2Bub1 facilitates genome-wide H3K4 trimethylation.

We next sought to characterize the relationship between decreased H3K4me3 and gene regulation following *Rnf40* deletion. The change in overall H3K4me3 abundance near the TSS displayed little correlation with the regulation of gene expression in response to *Rnf40* deletion (R = 0.208) (Additional file 1: Figure S2D). Recent studies showed that H3K4me3 peak width may play a potentially important function in gene transcription [24, 25]. Consistently, we observed that the width of H3K4me3 peaks is correlated to gene expression levels in MEF (Additional file 1: Figure S2E). Interestingly, H2Bub1 loss affected the width of H3K4me3 peaks much more than the height, thereby resulting in a significant narrowing of H3K4me3 peaks (Fig. 2c; Additional file 1: Figure S2F). In general, the summits of H3K4me3 peaks surrounding the TSS were shifted by ca. 50 bp towards the 5’ end of the gene after *Rnf40* deletion (Fig. 2c). Importantly, the downregulation of RNF40-dependent genes was associated with the narrowing of H3K4me3 peak width (R = 0.38) (Additional file 1: Figure S2G). In addition, RNF40-dependent genes (downregulated following *Rnf40* deletion) displayed significantly wider H3K4me3 peaks than those found on genes upregulated or unchanged (Fig. 2d). In comparison, H3K4me3 abundance on RNF40-dependent genes was significantly lower than unchanged genes (Fig. 2e). Consistent with the observed changes in gene regulation, the width of H3K4me3 peaks on downregulated genes was significantly shortened in *Rnf40*-deleted MEFs (Fig. 2f). In contrast to the specificity observed in H3K4me3 peak breadth, overall H3K4me3 abundance was significantly decreased irrespective of whether the associated gene was upregulated, downregulated, or unregulated in response to *Rnf40* deletion (Fig. 2g). Together these data indicate that transcriptional dependency on RNF40 is tightly coupled to the formation of broader H3K4me3 domains and less to overall H3K4me3 abundance.

Given the proposed importance of broad H3K4me3 domains for cell identity and function [24–27] and the correlation between changes in H3K4me3 peak breadth and H2Bub1, we next sought to examine whether the presence of broad H3K4me3 domains also correlated with changes in gene expression and was predictive for the requirement of RNF40 for gene expression. We therefore used the previously described approach [24, 25] to identify genes with top 5% broadest H3K4me3 domains, as well as similar numbers of genes displaying sharp H3K4me3 domains and random control genes in MEFs (Fig. 2h; Additional file 1: Figure S2H). In agreement with a recent report [25], broad H3K4me3 domains were correlated to gene expression (Fig. 2i). Importantly, loss of H2Bub1 resulted in a more significant narrowing of broad H3K4me3 peaks compared to sharp or random control H3K4me3 peaks (Fig. 2j; Additional file 1: Figure S2I–K). Moreover, the expression levels of genes displaying broad H3K4me3 peaks were significantly decreased in response to *Rnf40* deletion, whereas genes displaying sharp H3K4me3 peaks were moderately, but significantly, increased in their gene expression and randomly chosen control genes were unchanged in their expression (Fig. 2k). Taken together, we conclude that transcriptional dependency on H2Bub1 is closely coupled to the narrowing of H3K4me3 peak width.

### H2Bub1 selectively regulates transcription elongation rate and facilitates the spreading of H3K4me3 into the gene body

Release of RNA Polymerase II (Pol II) from transcriptional pausing is a crucial step in regulating the transcription of many genes. Recent findings documented that the spreading of H3K4me3 into the transcribed regions of active genes is inversely correlated to the Pol II pausing index [25]. Consistently, Pol II showed a clear enrichment on the gene body and 3’ end of genes with broad H3K4me3 domains in MEFs compared to genes with sharp H3K4me3 peaks and random control genes, while displaying no apparent differences in occupancy near the TSS (Additional file 1: Figure S3A). To further examine the association of H3K4me3 and transcriptional elongation, we examined the correlation between H3K4me3 and elongation rates using data available from HeLa cells [30]. Indeed, H3K4me3 peaks were significantly broader on genes displaying a high elongation rate (Additional file 1: Figure S3B), while there was no significant difference in the height of H3K4me3 peaks between genes displaying high and low elongation rates (Additional file 1: Figure S3C).

**Fig. 3:**
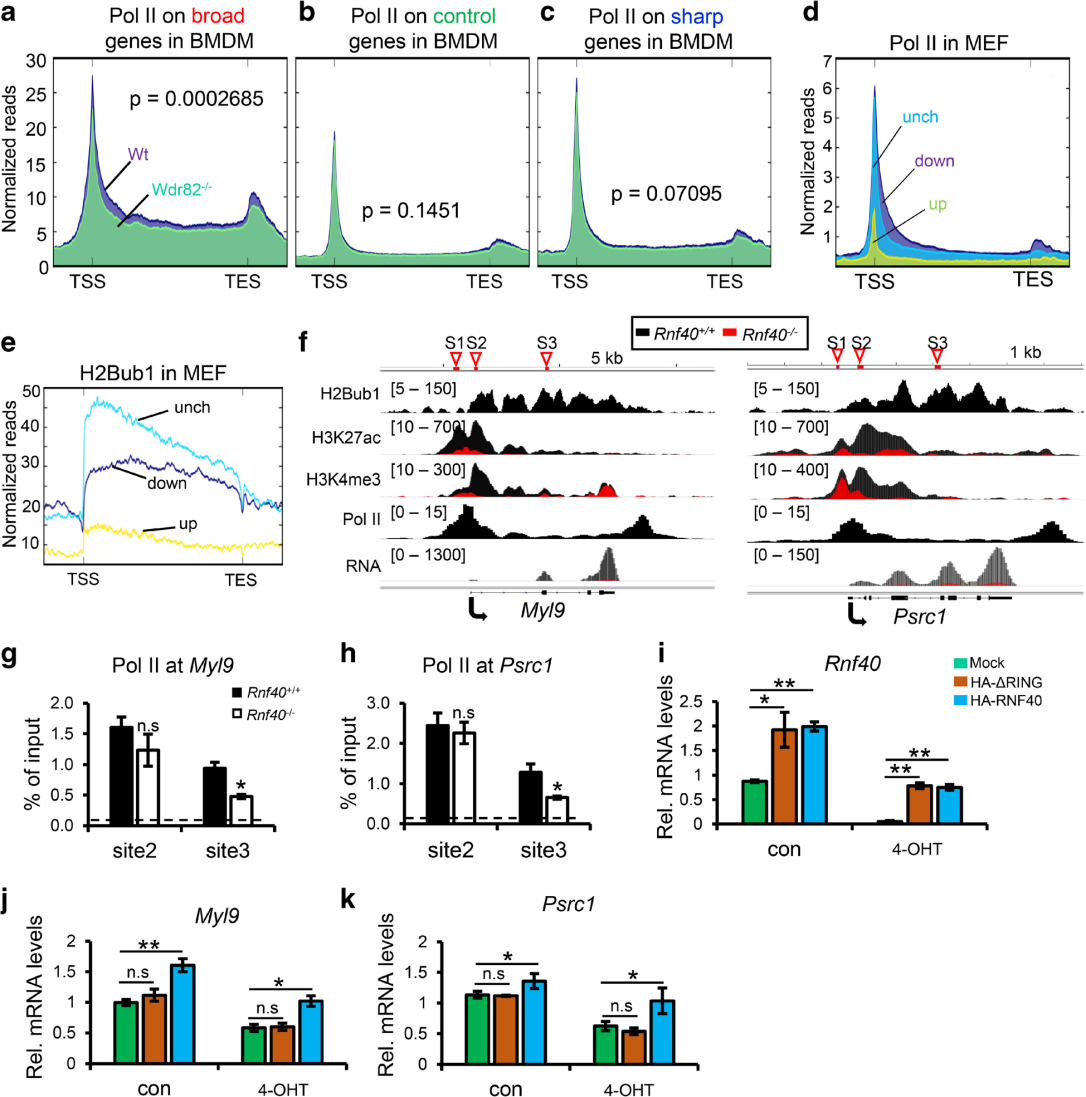
H2Bub1 and broad H3K4me3 are required for transcriptional elongation. **a**–**c** Comparison of the normalized occupancy of RNA Polymerase II (Pol II) in wild-type and *Wdr82*-null bone marrow-derived macrophage (BMDM) cells specifically on genes with broad, random control, and sharp H3K4me3 domains. *TSS* transcription start site, *TES* transcription end site. *p* value was calculated by unpaired Wilcoxon-Mann-Whitney-Test. **d**, **e** *Aggregate profiles* compare the normalized occupancy of Pol II (**d**) and H2Bub1 (**e**) in wild-type (*Rnf40* ^+/+^) MEFs from TSS to TES on genes upregulated (up), unchanged (unch), and downregulated (down) following *Rnf40* deletion. **f** The profiles show the occupancy of H2Bub1, H3K4me3, H3K27me3, H3K27ac, and Pol II as well as normalized RNA reads on the *Myl9* and *Psrc1* genes in *Rnf40* ^+/+^ (*black*) and *Rnf40* ^–/–^ (*red*) MEFs. **g**, **h** ChIP-qPCR for Pol II occupancy at different regions of the *Myl9* and *Psrc1* genes at the regions labeled in 3 F. *S1* site1, proximal to TSS, *S2* site2, downstream of TSS, *S3* site3, gene body. The data are shown as mean ± SD (*n* = 3). *p < 0.05; *n.s*. non-significant; unpaired two-tailed t-test. ChIP-qPCR for IgG was set as negative control and is indicated by the *dotted line*. **i**–**k** qRT-PCR analysis of *Rnf40*, *Myl9*, and *Psrc1* in mock, HA-RNF40-ΔRING, and HA-RNF40 transfected MEFs with or without 4-OHT treatment. *Mock* MEFs without vector transfection, *HA-RNF40-ΔRING* MEFs with RING finger-deleted *Rnf40* transfection, *HA-RNF40* MEFs with wild-type *Rnf40* vector transfection. The endogenous *Rnf40* gene was deleted by adding 250 nM of 4-OHT for five days. The data are shown as mean ± SD (n = 3). *p < 0.05; **p < 0.001; *n.s.* non-significant; unpaired two-tailed t-test

Although a positive correlation between H3K4me3 peak width and the transcription elongation machinery was reported in mESC [24], it remained possible that H3K4me3 may be a consequence of high elongation rate, rather than a cause. Therefore, we additionally analyzed the effect of the shortening of H3K4me3 peak width in *Wdr82*-null BMDM cells on the transcription elongation rate [31]. Loss of WDR82, a subunit of the SET1A/B complex catalyzing trimethylation of H3K4, resulted in a significant narrowing of H3K4me3 peaks at broad H3K4me3 genes, but not at genes with sharp H3K4me3 peaks and random control genes (Additional file 1: Figure S3E–G). In agreement with the correlation between broad H3K4me3 domains and increased elongation rate, deletion of *Wdr82* led to a significant decrease in Pol II occupancy on the gene body and 3’ end of genes with broad H3K4me3 domains, but not on genes displaying sharp H3K4me3 domains or random control genes (Fig. 3a–c). Thus, together these findings support a role for broad H3K4me3 domains in facilitating transcriptional elongation.

Next, we sought to examine whether H2Bub1 occupancy and transcription elongation rates are correlated on genes classified based on the width of the TSS-associated H3K4me3 domains. In contrast to a previous study which reported that H2Bub1 abundance is tightly coupled to transcription elongation rates [13], we found that genes displaying sharp H3K4me3 domains and low elongation rates (Additional file 1: Figure S3H) actually displayed the highest levels of H2Bub1 near the 5’ end while genes with broad H3K4me3 domains displaying high elongation rates showed significantly lower H2Bub1 occupancy near the 5’ end. Consistently, RNF40-dependent genes (which also display broader H3K4me3 domains, Fig. 2d) showed significantly higher Pol II enrichment (Fig. 3d) but lower H2Bub1 occupancy (Fig. 3e) across the gene body and 3’ end regions compared to unchanged genes. Therefore, these data indicate that the association between H2Bub1 occupancy and transcriptional elongation is intimately connected to H3K4me3 spreading into the gene body.

In order to further determine the role of H2Bub1 on transcriptional elongation, we analyzed individual RNF40-dependent genes to confirm the effects of RNF40 loss on gene expression and H3K4me3 occupancy. The *Myl9* and *Psrc1* genes displayed a clear enrichment of H2Bub1 across the gene body as well as broad H3K4me3 peaks near the TSS (Fig. 3f). Importantly, *Rnf40* deletion resulted in the narrowing of the TSS-associated H3K4me3 domain, decreased H3K27ac occupancy and *Myl9* and *Psrc1* mRNA levels. These effects were further confirmed by quantitative real-time polymerase chain reaction (qRT-PCR) and ChIP-qPCR for H2Bub1 and H3K4me3 (Additional file 1: Figure S3I–K). Consistent with a role of H2Bub1 in promoting transcriptional elongation, Pol II occupancy on the gene body, but not near the TSS, of the *Myl9* and *Psrc1* genes was significantly decreased in response to *Rnf40* deletion (Fig. 3g and h).

In order to confirm the specificity of the observed effects and the importance of RNF40-mediated ubiquitin ligase activity, we re-expressed wild-type RNF40 or a RING finger-deleted RNF40 in *Rosa26*-Cre^ERT2^, *Rnf40* ^loxP/loxP^ MEFs. *Rnf40* mRNA levels were restored to nearly normal levels in HA-ΔRING and HA-RNF40 MEFs after deleting the endogenous *Rnf40* gene (Fig. 3i). Importantly, re-expression of wild-type, but not ΔRING RNF40 was able to rescue RNF40-dependent gene expression (*Myl9* and *Psrc1*) (Fig. 3j and k), thereby confirming the specificity of the observed effects and reinforcing the importance of RNF40-mediated ubiquitin ligase activity in controlling the expression of RNF40-dependent genes.

### CDK9 is required for the establishment of broad H3K4me3 peaks and increased tissue-specific gene transcription via CDK9-RNF20/RNF40-H2Bub1 axis

**Fig. 4:**
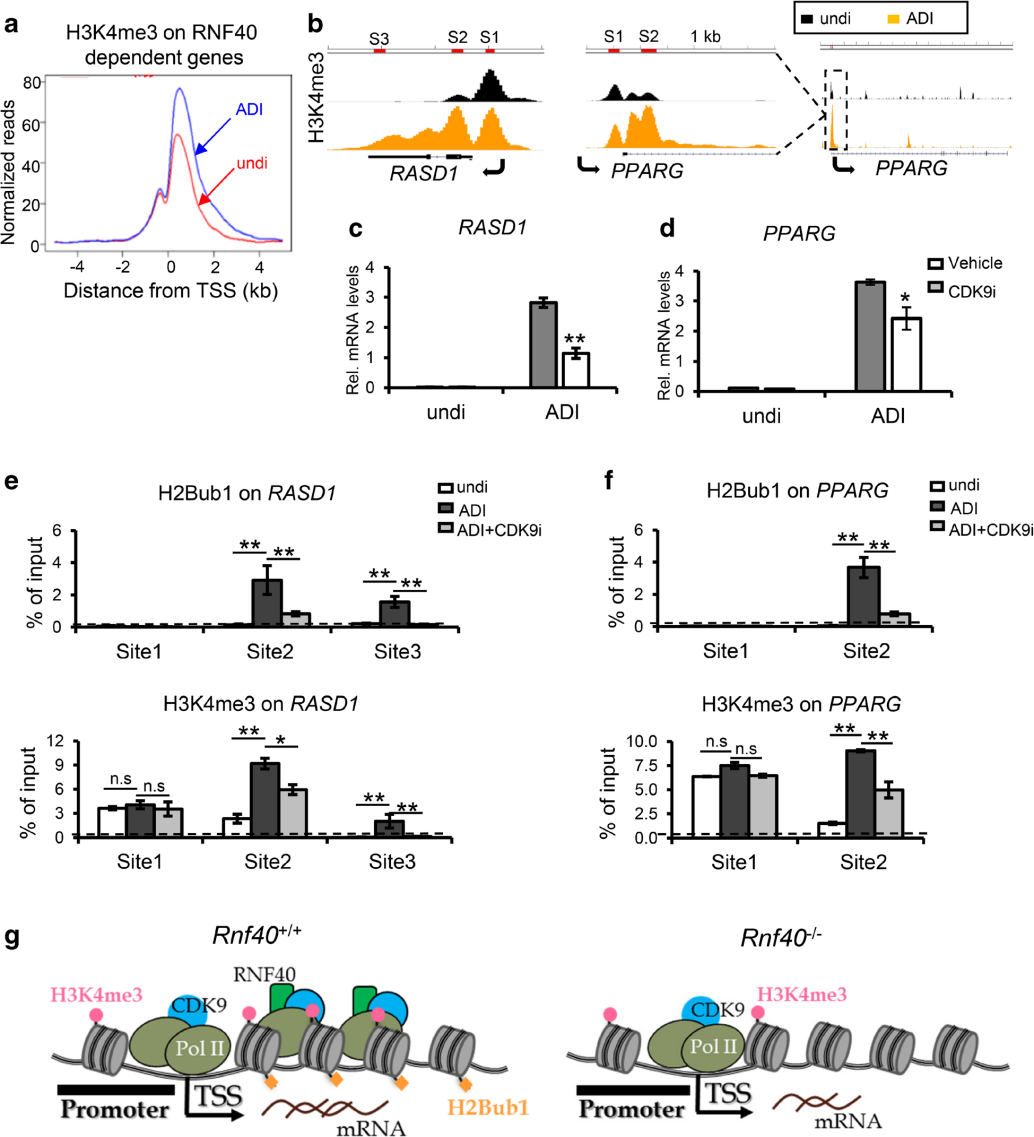
CDK9 is required for spreading of H3K4me3 into the body of tissue-specific genes. **a** *Aggregate profiles* display H3K4me3 occupancy on RNF40-dependent adipocyte-specific genes in undifferentiated (“un diff”) human mesenchymal stem cells (hMSC) and hMSC differentiated to the adipocyte (“ADI diff”) for five days. RNF40-dependent genes in hMSC differentiated to the adipocyte lineage for five days were selected from previously published data [23] based on log_2_-fold change in gene expression (siRNF40 vs.control siRNA) < –0.5. **b** ChIP profile for H3K4me3 on *RASD1* and *PPARG* in undifferentiated and adipocyte-differentiated hMSC. **c**, **d** qRT-PCR analysis of *RASD1* and *PPARG* during adipocyte differentiation (five days) with (CDK9i; 5 μM LDC000067 [32]) or without (con) CDK9 inhibitor treatment. Data are shown as mean ± SD (*n* = 3). *p < 0.05; **p < 0.001; *n.s.* nonsignificant; unpaired two-tailed t-test. **e**, **f** ChIP-qPCR analysis of H2Bub1 and H3K4me3 occupancy at different sites of *RASD1* and *PPARG* genes in “un diff,” “ADI diff,” and “ADI + CDK9i” cells. ADI + CDK9i, adipocytes differentiation for five days with CDK9 inhibitor treatment. Data are represented as mean ± SD (*n* = 3). *p < 0.05, **p < 0.001, unpaired two-tailed t-test. ChIP-qPCR for IgG is shown as negative control depicted as a *dotted line*. Examined regions are indicated in (**b**). **g** Model depicting how the CDK9-RNF40-H2Bub1 axis enhances the spreading of H3K4me3 into gene bodies to increase the transcription elongation rate of tissue-specific genes

Previous studies indicated that both H2Bub1 and broad H3K4me3 are associated with cell fate specification [23, 24, 26]. To understand the correlation between RNF40-dependent genes and broad H3K4me3 genes in a biological context, we performed Gene Ontology (GO) enrichment analysis for RNF40-dependent and broad H3K4me3 genes. Consistent with a role in cell fate specification, RNF40-dependent and broad H3K4me3-occupied genes were significantly enriched for developmental processes and cell cycle-related genes in MEFs (Additional file 1: Figure S4A and B). A further analysis of RNF40-dependent genes (log2 fold change < –1; *p* value < 0.05) displaying broad H3K4me3 domains (Additional file 1: Figure S4C) confirmed an enrichment for development-related genes (Additional file 1: Figure S4D). In agreement with this finding, RNF40-dependent adipocyte-specific genes identified in our previous study [23] also displayed a significant broadening of H3K4me3 peaks during adipocyte differentiation (Fig. 4a). This effect was confirmed at individual RNF40-dependent adipocyte-specific genes (*PPARG* and *RASD1*) (Fig. 4b, e, and f) and provides further support that broadening of H3K4me3 peaks may represent an important feature of RNF40-dependent cell fate-determining genes.

A previous study indicated that the spreading of H3K4me3 into the gene body is highly associated with the recruitment of the positive elongation machinery (CDK9, AFF4, ELL2, and TCEA1) [24]. Since H2B monoubiquitination depends directly upon CDK9 activity [9] and CDK9, the WAC adaptor protein, and RNF20/40 are similarly required for the induction of adipocyte-specific genes [23], we examined whether CDK9 is also required for the establishment of broad H3K4me3 domains on differentiation-specific genes using the newly reported CDK9-specific inhibitor LDC000067 [32]. Consistent with the effects elicited by siRNA-mediated CDK9 depletion [23], CDK9 inhibition led to a significant decrease in the induction of *PPARG* and *RASD1* during adipocyte differentiation (ADI) (Fig. 4c and d). In agreement with the reported importance of the CDK9-WAC-RNF20/RNF40 axis [23], H2Bub1 occupancy on *PPARG* and *RASD1* were significantly decreased in adipocytes following CDK9 inhibition (Fig. 4e and f). Importantly, H3K4me3 occupancy across the gene body, but not proximal to the TSS, was significantly decreased in response to CDK9 inhibition (Fig. 4e and f). Thus, we propose that the H2Bub1-H3K4me3 trans-histone pathway driven by the CDK9-RNF20/RNF40 axis is particularly important for cell lineage specification-associated broadening of H3K4me3 domains during cell fate specification (Fig. 4g).

### Increased gene expression in *Rnf40*-deficient MEFs is related to the loss of the *Ezh2* expression

**Fig. 5:**
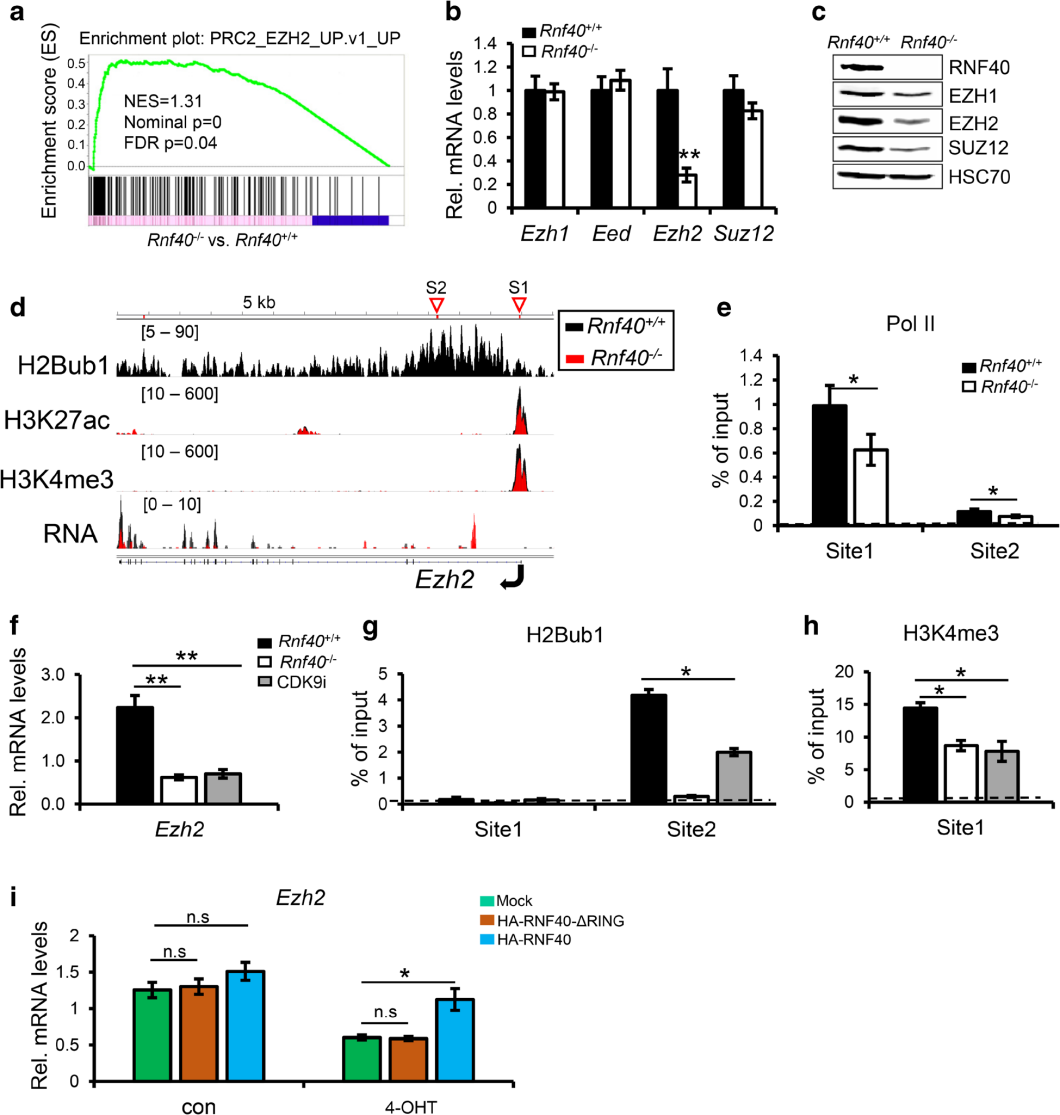
The CDK9-RNF40-H2Bub1 axis controls *Ezh2* expression. **a** GSEA of mRNA expression data reveal a significant enrichment of EZH2-suppressed genes (in TIG3 cells) in genes upregulated in *Rnf40* ^–/–^ MEFs. *NES* normalized enrichment score, *FDR* false discovery rate. **b** qRT-PCR analysis of PRC2 subunits *Ezh1*, *Ezh2*, *Eed*, and *Suz12* in *Rnf40* ^+/+^ and *Rnf40* ^–/–^ MEF cells. Data are shown as “relative mRNA levels,” mean ± SD (n = 3). *p < 0.05, **p < 0.001, n.s: *p* > 0.05, calculated with two-tailed unpaired t-test. **c** Western blot analysis of protein extracts from *Rnf40* ^+/+^ and *Rnf40* ^–/–^ MEFs using antibodies directed against RNF40, EZH2, SUZ12, and EZH1. HSC70 is shown as a loading control. **d** Profiles show the occupancy of H2Bub1, H3K27ac, H3K4me3, and expression counts (RNA) on the *Ezh2* gene in wild-type and *Rnf40*–null MEF. **e** ChIP-qPCR analyses of RNA Polymerase II (Pol II) occupancy near the TSS (site1) and in the gene body (site2) of the *Ezh2* gene. The PCR regions were labeled at (**f**). **f** qRT-PCR analysis of *Ezh2* mRNA levels in *Rnf40* ^+/+^, *Rnf40* ^–/–^, and CDK9 inhibitor-treated MEF. (**g**, **h**) ChIP-qPCR analysis of H2Bub1 and H3K4me3 occupancy near the TSS (site1) and in the gene body (site2) of the *Ezh2* gene in *Rnf40* ^+/+^, *Rnf40* ^–/–^, and CDK9 inhibitor-treated MEF. **i** qRT-PCR analysis of *Ezh2* mRNA levels with or without 4-OHT treatment in mock, HA-RNF40-ΔRING-, and HA-RNF40-expressing MEF

To examine whether loss of H2Bub1 impacts specific subsets of genes, we performed gene set enrichment analyses (GSEA) of mRNA-seq data and identified PRC2-suppressed (EZH2, SUZ12, and EED) and PRC1-suppressed genes as being significantly enriched in *Rnf40*-null cells (Fig. 5a; Additional file 1: Figure S5A and B). Strikingly, analysis of RNA-seq and H2Bub1 ChIP-seq data revealed H2Bub1 occupancy on the *Ezh2* gene and a selective decrease in its expression, while the expression of the remaining members of the PRC2 complex, which catalyzes H3K27 methylation, including *Suz12*, *Eed*, and *Ezh1*, was unaffected (Additional file 1: Figure S5C). These findings were confirmed by qRT-PCR (Fig. 5b). Consistent with an interdependence in their protein expression levels, western blot analysis of other PRC2 subunits revealed decreased protein levels not only of EZH2, but also for SUZ12 and EZH1 in *Rnf40* ^–/–^ MEFs (Fig. 5c). Moreover, this finding could be confirmed in various mouse tissues including the small intestine, colon, and lung following in vivo deletion of *Rnf40*, as well as in human mesenchymal stem cells (hMSC) and adipocytes following RNF40 knockdown (Additional file 1: Figure S5D–G). In addition, GSEA analysis confirmed that Polycomb-suppressed genes were significantly enriched in RNF40-depleted adipocytes (Additional file 1: Figure S5I) [23]. Consistently, deficiency of H2Bub1 resulted in a significant decrease in the active histone marks H3K4me3 and H3K27ac near the TSS of the *Ezh2* gene (Fig. 5d, g, and h). Furthermore, *Rnf40* deletion further led to a significant decrease of Pol II across the *Ezh2* gene (Fig. 5e). Interestingly, inhibition of CDK9 showed a similar effect as *Rnf40* deletion on *Ezh2* expression (Fig. 5f). The negative effect induced by the CDK9 inhibitor on *Ezh2* expression was further confirmed in hMSC and adipocytes (Additional file 1: Figure S5H). Consistently, inhibition of CDK9 led to a significant decrease of H2Bub1 occupancy across the *Ezh2* gene body and proximal H3K4me3 occupancy (Fig. 5g and h). Moreover, the decrease of *Ezh2* expression resulting from *Rnf40* deletion could be rescued by re-expressing wild-type but not the ΔRING form of RNF40 (Fig. 5i). Consistent with a previous report [33], cell cycle arrest by low serum treatment did not affect *Ezh2* mRNA levels, while deletion of *Rnf40* significantly decreased *Ezh2* expression both in low and normal serum cultured MEF (Additional file 1: Figure S5J–L). Therefore, we conclude that *Ezh2* expression is controlled by the CDK9-RNF20/RNF40-H2Bub1 axis in a cell cycle-independent manner.

**Fig. 6:**
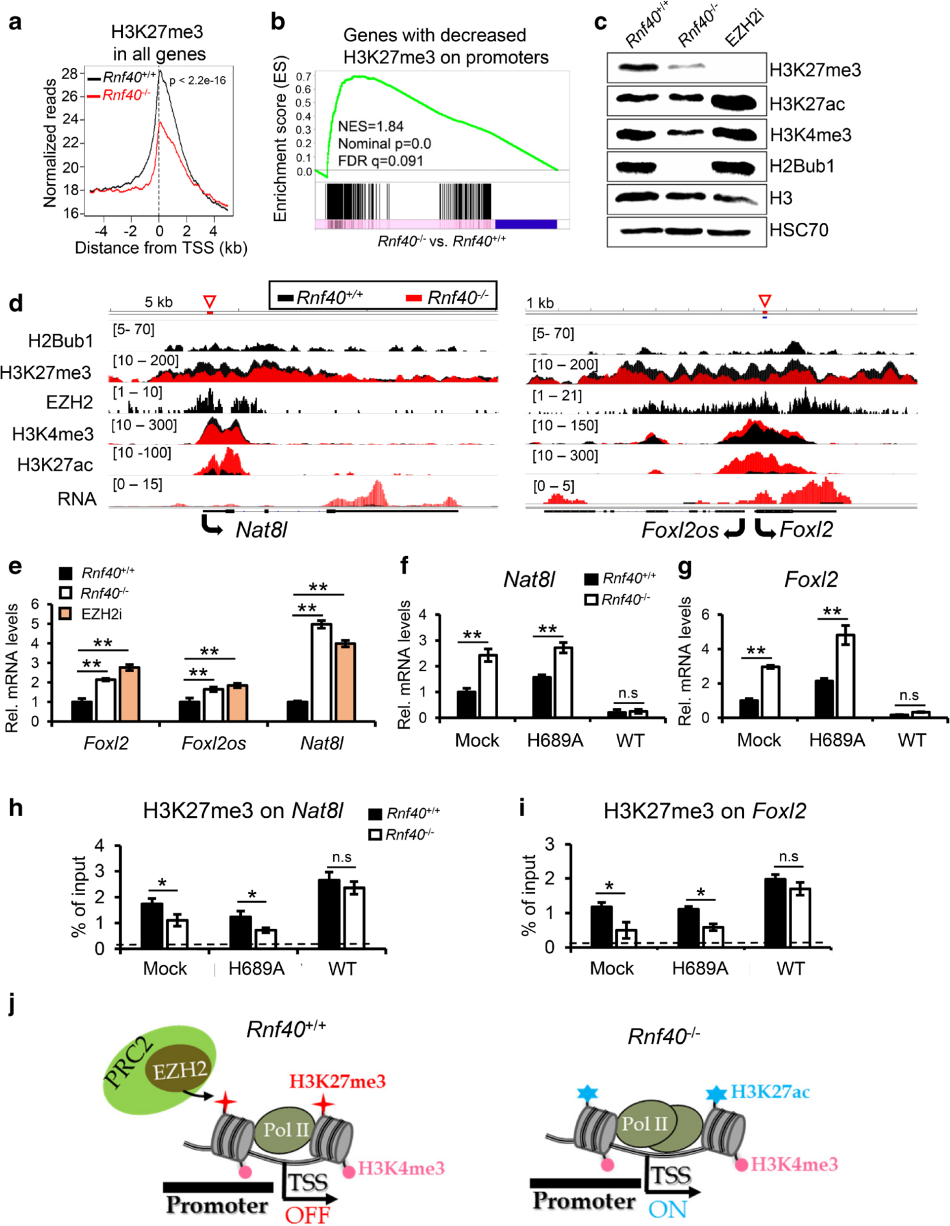
Decreased EZH2 expression is responsible for the upregulation of gene expression following *Rnf40* deletion. **a** Aggregate plot analysis of average H3K27me3 profiles surrounding TSS (±5 kb) in wild-type and *Rnf40* ^–/–^ MEFs. **b** GSEA shows a significant enrichment of genes upregulated in *Rnf40* ^–/–^ MEF in genes displaying decreased H3K27me3 surrounding the TSS. **c** Western blot analysis of whole protein extracts from DMSO as control (*Rnf40* ^+/+^; *Rnf40* ^–/–^) or EPZ6438-treated (*Rnf40* ^+/+^) MEFs (EZH2i) using antibodies against H3K4me3, H3K27me3, H3K27ac, H2Bub1, HSC70 (loading control), and H3 (loading control). Cells were treated with 1 μM EPZ6438 or DMSO as indicated for three days. **d** The profiles show the normalized occupancy of H2Bub1, H3K27me3, H3K4me3, H3K27ac, EZH2, and mRNA levels for the *Nat8l*, *Foxl2*, and *Foxl2os* genes in *Rnf40* ^+/+^ and *Rnf40* ^–/–^ MEFs. **e** qRT-PCR analysis of individual upregulated genes in *Rnf40* ^+/+^, *Rnf40* ^–/–^, and EZH2i-treated *Rnf40* ^+/+^ MEF cells. Data for qRT-PCR shows mean ± SD (*n* = 3); *p < 0.05, **p < 0.001, unpaired two-tailed t-test. **f**, **g** qRT-PCR analysis of *Nat8l* and *Foxl2* in control (mock), EZH2-H689A, and EZH2 (WT) expressing MEF with or without 4-OHT treatment. *Mock* control MEFs, *H689A* MEFs ectopically expressing a SET domain mutated EZH2, *WT* MEFs ectopically expressing wild-type EZH2. **h**, **i** ChIP-qPCR analysis of H3K27me3 occupancy near the TSS of the *Nat8l* and *Foxl2* genes in mock, EZH2-H689A, and WT EZH2 MEF with or without 4-OHT treatment. The investigated regions are indicated in (**d**). **j** Model describing the role of decreased *Ezh2* expression in controlling “RNF40-suppressed” genes

To understand the effects of decreased *Ezh2* expression on the distribution of H3K27me3 in *Rnf40*-null MEFs, we performed differential binding (DiffBind) analysis and identified 4372 genes occupied by H3K27me3 near the TSS (±1 kb), in which 97% (4241/4372) of those genes displayed a significant reduction in H3K27me3 occupancy following *Rnf40* loss (Additional file 1: Figure S6A), while H3K27me3-enriched distal intergenic regions were relatively unaffected (Additional file 1: Figure S6B). Aggregate plot analysis confirmed the significant decrease of H3K27me3 near the TSS (±5 kb) in *Rnf40*-null MEFs (Fig. 6a). In addition, we identified 861 EZH2 target genes (Additional file 2: Table S1) [34] and could demonstrate that H3K27me3 occupancy near the TSS (±5 kb) of these genes was significantly decreased in *Rnf40*-null MEFs (Additional file 1: Figure S6C).

We next sought to characterize the relationship between H3K27me3 occupancy near the TSS and the induction of gene expression following *Rnf40* deletion. Thus, we identified a gene set enriched for H3K27me3, which displayed a greater than two-fold decrease in H3K27me3 levels surrounding the TSS (Additional file 2: Table S1), and could observe that a large fraction of these genes was upregulated in *Rnf40*-deficient MEFs (Additional file 1: Figure S6D). Moreover, GSEA analysis using genes displaying decreased H3K27me3 occupancy in *Rnf40*-deficient MEFs further confirmed an enrichment of genes upregulated following *Rnf40* deletion (Fig. 6b). In addition, GSEA analysis of mRNA-seq data confirmed a significant enrichment of EZH2 target genes that were upregulated in *Rnf40*-null MEFs (Additional file 1: Figure S6E). Together these findings suggest that the deficiency of *Rnf40* and H2Bub1 results in a global decrease in H3K27me3 levels near the TSS of PRC2 target genes via decreased expression of *Ezh2*, thereby leading to a derepression of PRC2 target gene transcription.

In order to confirm that the observed effects were, indeed, due to decreased PRC2 activity, we compared the effects of treating *Rnf40* ^+/+^ MEFs with the EZH2 inhibitor EPZ6438 [35] to those observed following *Rnf40* deletion. The profiles for several PRC2 target genes (*Chd5*, *Nat8l*, *Kcnc3*, *Foxl2*, *Foxl2os*, and *Tgfa*; Fig. 6d, Additional file 1: Figure S6I) demonstrate that loss of RNF40 led to significant decreases in H3K27me3 occupancy at promoters, while H3K27ac occupancy and mRNA levels increased and H3K4me3 levels were largely unaffected. Consistent with the importance of this mechanism in vivo, *Rnf40* deletion in vivo also led to a significant increase of *Nat8l* expression (Additional file 1: Figure S6H). Consistent with the global decrease in H3K27me3 levels and a central role for decreased *Ezh2* expression in mediating increases in gene expression following *Rnf40* deletion (Fig. 6c), EZH2 inhibition significantly increased the expression of several PRC2 target genes (*Chd5*, *Nat8l*, *Kcnc3*, *Foxl2*, *Foxl2os*, and *Tgfa*) identified to be upregulated following the loss of *Rnf40* (Fig. 6e, additional file 1: Figure S6F), while non-PRC2-target genes (e.g. *Prsc1*) were unaffected by EZH2 inhibition (Additional file 1: Figure S6G). Importantly, increased expression of *Nat8l* and *Foxl2* resulting from *Rnf40* deletion could be rescued by re-expressing wild-type but not a methyltransferase-deficient mutant (H689A) of EZH2 (Fig. 6f and g) [36]. Moreover, ChIP-qPCR for H3K27me3 confirmed that overexpressing wild-type but not mutant EZH2 was sufficient to restore H3K27me3 occupancy on the *Nat8l* and *Foxl2* genes in the absence of RNF40 (Fig. 6h and i). Together these data suggest that the upregulation of a subset of genes following a loss of H2Bub1 elicited by *Rnf40* deletion can largely be attributed to a loss of *Ezh2* expression, rather than a direct repressive function of H2Bub1 (Fig. 6j).

### H2Bub1 coordinates homeobox gene activation and repression

**Fig. 7:**
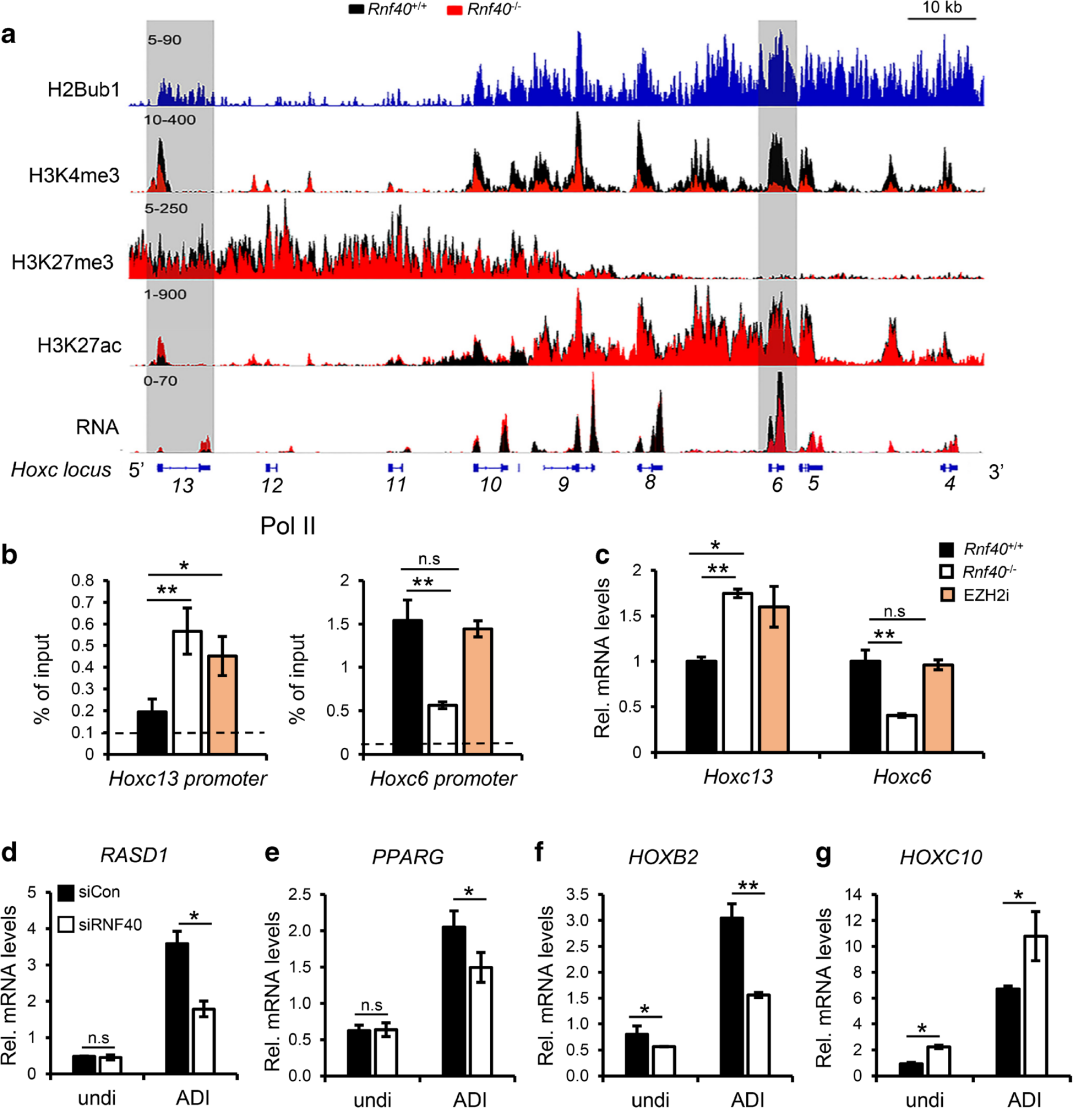
The *Hoxc* gene cluster as a prototype for RNF40/H2Bub1-dependent gene regulatory mechanisms. **a** The *Hoxc* gene cluster displays an enrichment of repressive modifications (H3K27me3) and low mRNA expression at the 5’ portion of the locus and active modifications (H3K4me3, H3K27ac) and higher mRNA levels at the 3' portion of the locus. **b** ChIP-qPCR analyses of RNA Polymerase II occupancy near the TSS of the *Hoxc13* and *Hoxc6* genes in the three samples. The data are shown as mean ± SD (n = 3). *p < 0.05; **p < 0.001; *n.s.* non-significant; unpaired two-tailed t-test. **c** qRT-PCR analysis of *Hoxc13* and *Hoxc6* in *Rnf40* ^+/+,^ *Rnf40* ^–/–,^ and EZH2i-treated *Rnf40* ^+/+^ samples (examined in 4D). Mean ± SD, *n* = 3; * *p* < 0.05; ** *p* < 0.001; *n.s.* non-significant; unpaired two-tailed t-test. **d**–**g** qRT-PCR analysis of the expression of the *RASD1*, *PPARG*, *HOXB2*, and *HOXC10* genes in control and adipocyte-differentiated hMSC transfected with non-targeting siRNA (sicon) and RNF40 siRNAs (siRNF40). qPCR was performed using the same samples examined in Additional file 1: Figure S5F

The *Hox* gene clusters represent prototypical evolutionarily conserved examples of coordinated transcriptional and epigenetic regulation during development controlled largely by H3K4me3 and H3K27me3. Importantly, H2Bub1 was previously reported to be required for the activation of some Hox genes [4]. Thus, the *Hox* genes represent an ideal opportunity to further examine the complexities of the differential effects of H2Bub1 loss on H3K4me3- and H3K27me3-dependent gene regulation at a single locus. As described in other systems, *Hox* clusters were decorated by both active and repressive histone modifications in our analyses (Fig. 7a). For example, the *Hoxc* cluster displayed increasing levels of the active histone marks H3K4me3 and H3K27ac in a 5’ to 3’ manner, while the repressive mark H3K27me3 displayed an inverse pattern (Fig. 7a). Consistent with the effects observed on global H3K4me3 levels, following *Rnf40* deletion and the loss of H2Bub1, the levels of H3K4me3 on the active 3’ portion of the cluster containing *Hoxc4*-*10* significantly decreased compared to other less active genes. Moreover, consistent with our other findings, H3K27me3 levels at the 5’ end of the cluster (e.g. *Hoxc9*-*13*) decreased. Finally, the levels of H3K27ac decreased at 3' *Hoxc* genes (*Hoxc4*-*8*) and increased at the 5' end of the cluster (*Hoxc10*-*13*) (Fig. 7a). ChIP-qPCR analysis confirmed that both loss of RNF40 or inhibition of EZH2 methyltransferase activity significantly decreased H3K27me3 levels near the TSS of the *Hoxc13* gene while H3K4me3 levels were low, but unaffected, and Pol II occupancy increased (Fig. 7b, left panel; Additional file 1: Figure S7A and B). In contrast, *Rnf40* deletion resulted in decreased H3K4me3 and Pol II occupancy on the *Hoxc6* gene, while EZH2 inhibition had no effect on their occupancy (Fig. 7b, right panel; Additional file 1: Figure S7A). Consistent with the changes in the dynamic equilibrium between active and repressive histone signatures on the *Hox* gene clusters caused by the absence of H2Bub1, the more 3’ *Hox* genes (e.g. *Hoxc6*) were downregulated following *Rnf40* deletion, but not EZH2 inhibition, while 5' genes (e.g. *Hoxc13*) were upregulated by either *Rnf40* deletion or EZH2 inhibition (Fig. 7c).

Given the important functions of Hox genes in stem-cell self-renewal and differentiation [37, 38], we also investigated two individual Hox genes (*HOXB2* and *HOXC10*) with different epigenetic contexts which are induced during adipocyte differentiation. As seen in ChIP-seq profiles for each gene (Additional file 1: Figure S7C), the TSS of both *HOXB2* and *HOXC10* were significantly enriched for H3K4me3 near the TSS, while *HOXC10* also displayed significant H3K27me3 occupancy upstream of the TSS. Consistently, the expression of both *HOXB2* and *HOXC10* was significantly increased during adipocyte differentiation. This effect coincided with increased occupancy of H3K4me3 on the *HOXB2* gene and decreased occupancy of H3K27me3, but no significant change of H3K4me3 on the *HOXC10* gene during adipocyte differentiation (Additional file 1: Figure S7C). Consistent with the epigenetic context of both genes and the differential effects of RNF40 loss on Polycomb-repressed and H3K4me3-dependent genes, *RNF40* depletion led to downregulation of the H3K4me3 occupied *HOXB2* gene and upregulation of the H3K27me3 and H3K4me3 co-occupied *HOXC10* gene (Fig. 7f and g). Together, these data suggest that H2Bub1 differentially regulates *Hox* genes in a context-dependent manner by coordinating the equilibrium between active (H3K4me3 and H3K27ac) and repressive (H3K27me3) histone modifications not only in MEFs, but also in a biologically relevant differentiation system.

### Transcriptional activation of a subset of RNF40-suppressed genes is associated with enhancer activation

**Fig. 8:**
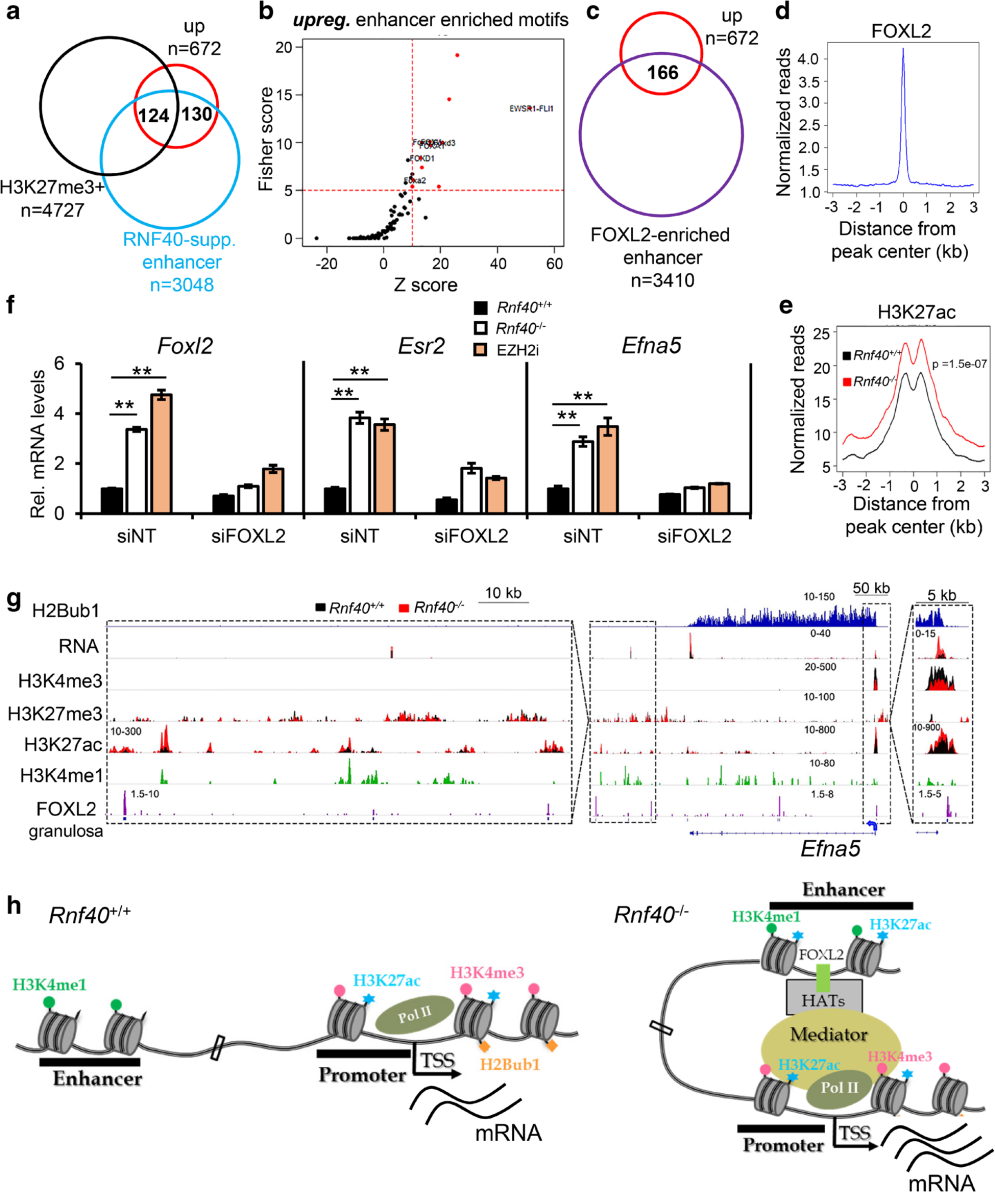
The upregulation of a subset of RNF40-suppressed genes is associated with the activation of FOXL2-bound enhancers. **a** *Venn diagram* displaying the overlap between enhancers activated following RNF40 loss (“RNF40-supp. enhancer”), H3K27me3-enriched genes (H3K27me3+), and genes upregulated following *Rnf40* deletion (up). RNF40-suppressed enhancers were obtained by DiffBind analysis (see Additional file 1: Figure S8D) and selected based on increased H3K27ac at intergenic regions (fourfold increased H3K27ac occupancy in *Rnf40* ^–/–^ MEF, FDR < 0.05). RNF40-suppressed enhancer associated genes were identified by performing GREAT analysis of the significantly increased enhancers. **b** *Plot* displaying the Fisher and Z Scores of motif analysis performed using the 254 gene-associated enhancers shown in *A*. The Fisher Score >5 & Z Score > 10 indicates significantly enriched motifs (shown in *red*). **c** *Venn diagrams* showing the numbers shared by upregulated genes and FOXL2-enriched enhancer associated genes. FOXL2-enriched enhancer-associated genes were obtained by GREAT analysis of FOXL2-enriched enhancers (from Additional file 1: Figure S8G). **d**, **e** Aggregate profiles show FOXL2 average signal surrounding the center (±3 kb) of FOXL2-bound peaks displaying increased H3K27ac signals in *Rnf40* ^+/+^ or *Rnf40* ^–/–^ MEFs (the 166 gene-associated enhancers from C). The *p* value (**e**) was calculated by unpaired Wilcoxon-Mann-Whitney test. **f** qRT-PCR analysis of *Foxl2*, *Esr2*, and *Efna5* genes in *Rnf40* ^+/+,^ *Rnf40* ^–/–,^ and EZH2i-treated *Rnf40* ^+/+^ MEFs transfected with non-targeting siRNA (siNT) and *Foxl2* specific targeting siRNA treatment. **g** The ChIP-seq profiles for H2Bub1, H3K4me3, H3K27me3, H3K27ac, FOXL2, and H3K4me1 on the *Efna5* gene. **h** Model describing the role of increased *Foxl2* expression in enhancer and gene activation following RNF40 loss

As described above, a number of genes were upregulated after *Rnf40* deletion. While some of this regulation could be attributed to decreased H3K27me3 occupancy near the TSS, up to 41% (276/672) of upregulated genes displayed no significant H3K4me3 or H3K27me3 occupancy (Additional file 1: Figure S8A). Therefore, we examined whether these genes may be regulated via H2Bub1-dependent regulation of distal enhancer activity. We identified 30,893 active enhancers (H3K27ac^+^/H3K4me1^+^/H3K4me3^−^ regions) by combining H3K4me1 ChIP-seq data in MEFs [39] (Additional file 1: Figure S8B and C) and performing differential occupancy (DiffBind) analysis to identify H3K27ac-enriched enhancer regions which change between *Rnf40* wild-type and null MEFs. These analyses uncovered 3335 regions displaying a significant increase in H3K27ac occupancy (log_2_-fold change (*Rnf40* ^+/+^ versus *Rnf40* ^–/–^) < -2, false discovery rate (FDR) < 0.05) and 2362 enhancers displaying significantly decreased H3K27ac levels (log_2_-fold change > 2, FDR < 0.05) following *Rnf40* deletion (Additional file 1: Figure S8D, Additional file 3: Table S2). Interestingly, more than 38% (254/672) of upregulated genes were associated with enhancers displaying significantly increased H3K27ac occupancy in *Rnf40* ^–/–^ MEFs, but no significant enrichment of H3K27me3 at these sites in either condition (Fig. 8a; Additional file 1: Figure S 8E). However, only 14.2% (114/802) of downregulated genes displayed significant decreases in H3K27ac occupancy at enhancers in *Rnf40* ^–/–^ MEFs (Additional file 1: Figure S8F). Hence, we hypothesized that the upregulation of a subgroup of RNF40-suppressed genes is independent of a direct effect on PRC2 and may instead be associated with the de novo activation of distal enhancers.

To uncover potential transcription factors which may contribute to enhancer activation and upregulation of genes in *Rnf40* ^–/–^ MEFs, we performed a sequence-based motif analysis of the upregulated gene-associated enhancers and identified a significant enrichment of Forkhead box protein binding motifs (Fig. 8b). Consistent with our observation that the expression of *Foxl2* was significantly increased in *Rnf40* ^–/–^ MEFs (Fig. 6e), we identified 3223 enhancers in our study which were occupied by FOXL2 in a published ChIP-seq dataset [40] (Additional file 1: Figure S8G). In addition, GREAT analysis of those regions identified the FOXL2-enriched enhancer-associated genes, which contained more than 25% (166/672) of the upregulated genes (Fig. 8c) and 100 genes (more than 60%) which were upregulated and displayed enhancer activation following *Rnf40* deletion (Additional file 1: Figure S8H). Consistent with increased enhancer activation, the H3K27ac occupancy surrounding these FOXL2-enriched distal regions was significantly increased in *Rnf40* ^–/–^ MEFs (Fig. 8d and e).

In order to confirm the role of FOXL2 in the upregulation of this subset of genes in *Rnf40* ^–/–^ MEFs, we examined the effects of siRNA-mediated FOXL2 depletion in MEFs following *Rnf40* deletion. Consistent with a previous study demonstrating the importance of FOXL2 for their expression [40], we observed that both the *Esr2* and *Efna5* genes were significantly upregulated following *Rnf40* deletion as well as following EZH2 inhibitor treatment (Fig. 8f). Furthermore, FOXL2-targeted genes (*Esr2* and *Efna5*) were increased in the ovary of a *Rnf40*-deleted mouse (Additional file 1: Figure S8I). Importantly and consistent with an indirect effect mediated by FOXL2, these effects were blocked by FOXL2 depletion (Fig. 8f). Moreover, ChIP-seq profiles confirmed that H3K27ac occupancy on each of those genes was increased at FOXL2-bound enhancers following *Rnf40* deletion (Fig. 8g; Additional file 1: Figure S8J). Together these data support a central role for FOXL2 in mediating enhancer activation and increased gene expression of a subset of genes whose expression increases following *Rnf40* deletion (Fig. 8h).

## Discussion

The complex regulatory network of post-translational histone modifications has long been hypothesized to play a significant role in controlling the timely activation or repression of gene transcription [41]. Here we investigated the genome-wide occupancy of H2Bub1 and examined the effects of its loss following *Rnf40* deletion on other post-translational histone modifications at proximal and enhancer regions, and investigated the relation of these alterations to changes in gene transcription. In addition to providing a genome-wide confirmation of the previously reported H2Bub1-H3K4me3 trans-histone tail crosstalk [42, 43], our work describes a previously unknown role for RNF40-mediated H2B monoubiquitination in the establishment and maintenance of broad H3K4me3 domains, which appear to selectively promote the transcriptional elongation of tissue-specific genes. In addition, we provide the first mechanistic explanation by which the loss of RNF40 can lead to increased gene expression. Specifically, a subset of PRC2-repressed genes is upregulated following *Rnf40* deletion via decreased *Ezh2* expression and a resulting decrease in H3K27me3 and a concordant increase in H3K27ac occupancy near their TSSs (Fig. 6j). We also identified an additional group of “RNF40-suppressed genes,” which is associated with increased enhancer activity via upregulation of *Foxl2* gene expression (as a consequence of decreased *Ezh2* expression and PRC2 activity) in *Rnf40*-null MEFs (Fig. 8h).

### RNF40-regulated genes display low and moderate H2Bub1 occupancy

In order to obtain efficient activation of gene transcription, the signals enabling transcriptional activity, including active histone modifications, appear to require a certain threshold in order to facilitate gene expression [44]. According to our data, genes which display the highest occupancy of H2Bub1 and other active histone modifications appear to be more robustly expressed and less sensitive to changes in the presence of individual histone modifications. We hypothesize that, even in the absence of H2Bub1, these genes retain sufficient additional active signals to maintain high levels of transcription. In contrast, inactive or lowly active genes, such as poised genes marked by the PRC2 complex, may require higher levels of additional activation signals to switch from a repressed to an active state and therefore be more dependent upon individual histone modifying enzymes. Furthermore, there seems to be a complex regulatory mechanism acting on genes marked by varying degrees of both active and repressive histone modifications as we observed for low to moderate H2Bub1-occupied genes, whose transcription highly depends upon changes in histone modifications facilitated by the recruitment of tissue-specific transcription factors [44]. Thus, this class of genes appears to be particularly vulnerable to changes in expression elicited by the loss of either active or repressive marks.

### RNF40-mediated H2Bub1 governs H3K4me3 peak width to increase transcription elongation rate

Consistent with the H2Bub1-H3K4me3 trans-histone crosstalk model in which H2Bub1 facilitates the trimethylation of H3K4 by the SET/COMPASS complex [45], our study shows that the absence of H2Bub1 results in a decrease, but not a total loss, of H3K4me3 levels genome-wide. Notably, the decrease in H3K4me3 occupancy was most apparent at regions downstream of the TSS, which were also occupied by H2Bub1. Following loss of H2Bub1, the width of H3K4me3 peaks was affected much more than their height, thereby resulting in a significant narrowing of the peaks toward the TSS. We speculate that the bulk of H3K4me3 near the TSS may be catalyzed by SET/COMPASS or other H3K4 methyltransferases in an RNF40/H2Bub1-independent manner, but that transcriptional elongation-associated spreading of H3K4me3 into the gene body is highly dependent upon RNF20/40-mediated H2B monoubiquitination. This effect can also be observed on the *Hoxc* gene cluster where H3K4me3 on each of the *Hoxc* genes decreases, but some degree of H3K4me3 remains and becomes more focused around the TSS. These effects closely resemble those observed in *Mll1*- deficient MEFs [46], suggesting that H2Bub1 may be capable of directing MLL-dependent H3K4 methylation downstream of the TSS.

Although H2Bub1 had broad effects on H3K4me3 occupancy genome-wide, the H2Bub1-H3K4me3 crosstalk selectively regulated a subset of genes in response to *Rnf40* deletion. We further determined that transcriptional dependency on H2Bub1 is highly linked to the narrowing of H3K4me3 peak width. Consistent with the finding that increasing H3K4me3 width is associated with increased transcription elongation rates [25], RNF40-dependent genes show broader H3K4me3 peaks and higher transcription elongation rates compared to RNF40-independent and RNF40-suppressed genes. Moreover, given that the width of H3K4me3 peaks is highly dependent on H2Bub1, the transcription of genes with the broad H3K4me3 domains depended on RNF40 more than genes with sharp H3K4me3 peaks near the TSS and random control genes.

Previous work from the Oren lab reported that high H2Bub1 occupancy is associated with high transcription elongation rates [13], but that RNF20 depletion did not affect elongation rates of RNF20-dependent genes [47]. However, in this work we examined the context-dependency of the effects of RNF40/H2Bub1 loss on transcription and observed that RNF40-dependent genes with broader H3K4me3 and higher elongation rates showed significantly lower occupancy of H2Bub1 across the gene body than RNF40-independent genes with narrower H3K4me3 peaks and lower elongation rates. Given the correlation between the positive elongation machinery and H3K4me3 width [24], we suggest that the function of H2Bub1 in facilitating transcriptional elongation is closely linked to the RNF40-dependent broadening of H3K4me3. Although our work has revealed that H2Bub1 is essential for broadening H3K4me3 peaks, it remains to be determined how H2Bub1 selectively facilitates the broadening of a subset of genes. We hypothesize that some regulators may function to prevent the spreading of H3K4me3 domain. While changes in the activity or recruitment of specific methyltransferases such as UpSET in *Drosophila melanogaster* or MLL5 in humans may explain these effects [25], another possibility would be the specific recruitment or exclusion of the RACK7 histone demethylase complex, which has been shown to suppress the broadening of H3K4me3 at promoters and enhancers [48].

Recent studies uncovered a previously unrecognized association of broad TSS-associated H3K4me3 domains with the expression of tumor suppressor and cell identity genes [24, 25]. Consistent with potential tumor suppressor functions of RNF20/40 and H2Bub1 and their requirement for stem cell differentiation [17, 23], we observed a widening of H3K4me3 peaks on RNF40-dependent lineage-specific genes during adipocyte differentiation. Consistently, we previously demonstrated that CDK9-WAC-RNF20/RNF40-directed H2B monoubiquitination is required for tissue-specific gene transcription [23]. Moreover, we demonstrate that the activity of CDK9, the catalytic component of the Positive Transcription Elongation Factor-b complex, is required for the spreading of H3K4me3 peaks into the gene body of RNF20/40-dependent differentiation-induced genes. Additionally, and consistent with a direct role of broad H3K4me3 domains in facilitating transcriptional elongation, *Wdr82* deletion in BMDM cells resulted in a shortening of H3K4me3 peaks [31] and decreased the transcription elongation rate specifically at genes with broad H3K4me3 domains. Therefore, we suggest that CDK9, RNF20/RNF40, H2Bub1, and broad H3K4me3 domains cooperatively facilitate transcriptional elongation of tissue-specific genes. Given that broad H3K4me3 domains are a particular epigenetic hallmark of cell identity and tumor suppressor genes [24, 25], further studies on the CDK9-RNF20/RNF40-H2Bub1-broad H3K4me3 axis to determine the functional interconnectivity between WDR82, H2Bub1, and transcriptional elongation will likely reveal important insight into the epigenetic regulatory mechanisms controlling important processes such as embryogenesis and tumorigenesis. Moreover, the extent and molecular mechanisms by which broad H3K4me3 domains and H2Bub1 promote transcriptional elongation as well as the specific mechanisms leading to spreading of H3K4me3 at only a subset of genes remain to be determined.

### Loss of H2Bub1 causes de-repression of a subset of genes

Consistent with findings following RNF20 knockdown [11], we find that the vast majority of “RNF40-suppressed” genes do not display significant levels of H2Bub1, thereby suggesting that their regulation may occur through more indirect mechanisms. Consistently, we find that the *Ezh2* gene, encoding the catalytic component of the PRC2 complex, which mediates H3K27 methylation, displays a significant level of H2Bub1 occupancy and requires RNF40 for its full expression. The regulation of RNF40-mediated H2Bub1 on *Ezh2* expression is not isolated to cultured MEF cells, but was confirmed in human cell lines and various tissues from a global conditional in vivo *Rnf40* knockout mouse model.

Furthermore, consistent with a central role for EZH2 as a central mediator of H2Bub1-dependent “gene repression,” small molecule inhibition of EZH2 activity resulted in a similar derepression of H3K27me3-targeted genes which were upregulated in *Rnf40*-deficient MEFs. The expression of this subset of PRC2-regulated genes is likely related to a dynamic antagonism between H3K27me3 and H3K27ac at p300/CBP and PRC2 co-targeted sites [49–51]. Thus, the upregulation of “RNF40-suppressed” genes appears to be related to a shift in the balance between H3K27me3 and H3K27ac, whereby decreased H3K27 methylation enables the acetylation of the same residue at these loci.

In addition to direct PRC2-regulated genes, we also observed the upregulation of genes which demonstrated increased activity of nearby enhancers. This increased enhancer activity was associated with the derepression of *Foxl2* as a consequence of *Ezh2* downregulation in *Rnf40* ^–/–^ MEFs. Interestingly, the promoter of the *FOXL2* gene was found to be hypermethylated in ovary granulosa cell tumors concomitant with increased EZH2 expression [52]. However, whether these effects coincide with changes in RNF40 activity or H2Bub1 occupancy in ovary granulosa cell tumorigenesis remains unknown.

We further determined that genes upregulated following *Rnf40* deletion were enriched for developmental regulators, further supporting a critical function of RNF40 in directing cell fate determination. Consistent with a context-dependent function of H2Bub1 in regulating different groups of genes, while we previously demonstrated a central role for RNF20/40-dependent H2B monoubiquitination in differentiation to the osteoblast and adipocyte lineages [23], another group reported that H2Bub1 levels decrease during myoblast differentiation [53]. Interestingly, while the regulatory role of RNF40 in ovarian development remains to be determined, the FOXL2-regulated genes *Esr2* and *Enf5a*, which are required for ovary development [40], were increased in the ovary following global conditional *Rnf40* deletion. Thus, it is possible that the RNF20/40-H2Bub1 pathway may promote cell differentiation to one lineage and suppress that of another lineage in a given epigenetic context while promoting differentiation to other lineages in a different context.

## Conclusions

In conclusion, we provide evidence and insight into the apparent discrepancy between the association of H2Bub1 with active gene transcription and the unexpected finding that a nearly equal fraction of genes are up- or downregulated following its loss. Our results support a model in which the direct function of RNF40-mediated H2B monoubiquitination increases transcriptional elongation by promoting the spreading of H3K4me3 into the 5’ transcribed region of a select subgroup of genes. However, given the finding that the *Ezh2* gene is a major target of RNF40 and H2Bub1, and the demonstration that the effects of *Rnf40* deletion on these “H2Bub1-suppressed” genes can be mimicked by inhibition of EZH2 catalytic activity, our data support a model in which “suppression” of gene transcription by H2Bub1 is mediated via indirect effects through PRC2. These findings, together with our results supporting a role for H2Bub1 in controlling H3K4me3 on RNF40-dependent genes, provide important insight into the enigmatic role of H2Bub1 in transcription. Further studies examining the effects of *Rnf40* deletion in additional cell types and tissues, in conjunction with in vivo disease models, will shed further light into the biological and mechanistic functions of H2Bub1 and further elucidate its context-dependent function.

## Methods

### Conditional *Rnf40* knockout mouse model and isolation of inducible knockout MEFs

All animal work was performed in agreement with the Institutional Animal Care and Use Committee and the Institutional Guidelines for Humane Use of Animals in Research. The construct for generating conditional *Rnf40* knockout mice was generated using a construct containing two loxP sites surrounding exons 3 and 4 of the *Rnf40* gene and a neomycin selection cassette was surrounded by two short flippase recognition target (FRT) sites. The targeting construct was electroporated in MPI II ES cells and targeted clones were identified by quantitative and long-range PCR. Following the generation of chimeras and verification of germline transmission, the neomycin cassette was removed to generate *Rnf40* ^loxP^ mice by crossing to a transgenic mouse line expressing the FLP recombinase in all tissues [54]. The *Rnf40* ^loxP^ mice were next crossed to a transgenic line expressing a tamoxifen-inducible Cre recombinase (Cre^ERT2^) inserted into the ubiquitously expressed *Rosa26* locus [29]. The inducible *Rnf40* knockout MEFs were obtained by intercrossing *Rosa26*-Cre^ERT2^, *Rnf40* ^loxP/wt^ mice. Finally, MEFs were isolated from 13.5 postcoitum mouse embryos as previously described [55] and homozygous *Rosa26*-Cre^ERT2^, *Rnf40* ^loxP/loxP^ embryos were utilized to generate MEFs.

In order to examine the effects of in vivo *Rnf40* deletion, two *Rosa26*-Cre^ERT2^, *Rnf40* ^loxP/loxP^ and *Rnf40* ^wt/wt^ mice were treated with tamoxifen every other day. 5% Tamoxifen (w/v; Sigma-Aldrich) was dissolved in ethanol and subsequently mixed 1:10 with sunflower oil and injected intraperitoneally every other day for one week with a total dose of 1.5 mg tamoxifen per day. Weight and general health status were monitored daily and two weeks after commencing injections various tissues were harvested.

### Cell culture

Primary MEFs were cultured in high-glucose GlutaMAX™-DMEM (Invitrogen) supplemented with 10% FBS Superior (Biochrom), 1% penicillin–streptomycin (Sigma-Aldrich), and 1% non-essential amino acids (Invitrogen) at 37 °C, 5% CO_2_. For deletion of the conditional *Rnf40* allele, MEFs were passaged in growth medium supplemented with 250 nM of 4-hydroxytamoxifen (4-OHT). After five days, cells were grown for another three days in the absence of 4-OHT. Where indicated, *Rnf40* ^+/+^ MEFs were treated with 1 μM EPZ6438 (Selleck Chemicals) for three days and forward and reverse transfection of siRNA were performed in *Rnf40* ^+/+^ and *Rnf40* ^–/–^ MEFs using RNAiMAX (Thermo Scientific) according to the manufacturer’s instructions. Non-targeting siRNA (D-001210-05-50, Dharmacon) was used as a negative control. Targeted mouse Foxl2 SMARTpool siRNAs (L-043309-01-0005, Dharmacon) contained the sequences 5’-GCGCAGUCAAAGAGGCCGA-3’, 5’-ACUCGUACGUGGCGCUCAU-3’, 5’-UAGCCAAGUUCCCGUUCUA-3’, and 5’-CGGGACAACACCGGAGAAA-3’.

The constructs for overexpressing wild-type *Rnf40*, RING finger-deleted *Rnf40* (ΔRING-RNF40), wild-type *Ezh2*, and SET domain-mutated *Ezh2* (H689A) were generated by cloning *Rnf40*, ΔRING-RNF40, *Ezh2*, and H689A PCR products into a pSG5-HA-hygromycin vector in which the expressed HA-tagged fusion protein and hygromycin resistance gene were expressed from a single open reading frame and separated by a P2A sequence resulting in the production of two separate polypeptides. The primers for cloning are listed in Additional file 4: Table S3. Expression constructs were transfected into *Rosa26*-Cre^ERT2^, *Rnf40* ^loxP/loxP^ MEFs using Lipofectamine 2000 (Thermo Fisher Scientific). Four days after transfection, hygromycin-resistant MEFs were selected by using 300 μg/mL of hygromycin for approximately two months.

hMSC-Tert cells [56] were passaged in phenol red-free low-glucose MEM (Invitrogen) supplemented with 10% FBS and 1% penicillin–streptomycin (Sigma-Aldrich) at 37 °C, 5% CO_2_. For adipocyte differentiation [23], hMSC were cultured in MEM supplemented with 15% FBS, 2 × 10^−6^ M insulin, 0.45 mM isobutylmethyl-xanthine, 10^−5^ M troglitazone, and 10^−4^ M dexamethasone. For knockdown of RNF40, hMSC were transfected with RNF40 [23] or non-targeting siRNA (D-001210-05-50, Dharmacon) using RNAiMAX (Thermo Scientific) 16 h prior to the induction of adipocyte differentiation according to the manufacturer’s instructions. For inhibiting CDK9, hMSC were treated with 5 μM of LDC000067 2 h prior to the induction of adipocyte differentiation.

### Western blotting and qRT-PCR

Protein extraction, western blot analysis, RNA isolation, reverse transcription, and qPCR were performed as previously described [57]. The primary antibodies for western blot and primers for qRT-PCR are listed in Additional file 4: Table S3 and Additional file 5: Table S4, respectively.

### ChIP, ChIP-qPCR, ChIP-seq, and RNA-seq library preparation

ChIP for histone modifications was performed as described using 10 min crosslinking [58] or 15 min for RNA Polymerase II. Antibodies for ChIP and their dilutions are listed in Additional file 5: Table S4. ChIP qRT-PCR analysis of the occupancy of Pol II, H2Bub1, H3K4me3, and H3K27me3 was performed as described previously [23]. Immunoprecipitated DNA of each sample was quantitated using a Qubit 3.0 (Life technologies). A total of 5 ng of DNA was sonicated to obtain 200 bp fragments using the Bioruptor® Pico (Diagenode) and sequencing libraries were prepared using the NEBNext Ultra DNA library preparation kit according to the manufacturer’s protocol (New England Biolabs).

Total RNA was isolated from wild-type and *Rnf40*-null MEF cells at passage 3. Total RNA quality was verified using a Bioanalyzer 2100 (Agilent) and libraries were prepared from 1 μg of total RNA using the NEXTflex™ Rapid Directional RNA-Seq Kit according to the manufacturer’s protocol (Bio Scientific).

Each RNA and ChIP DNA library was quantified using a Qubit 3.0 (Life Technologies) and fragment sizes was checked using the Bioanalyzer 2100 (Agilent). Finally, 75 bp single-end sequencing for H3K4me3 and 51 bp single-end sequencing for other histone modifications were performed with single indexing using the NextSeq or HiSeq 2500 (Illumina) platforms, respectively, as described before [12]. ChIP-seq and RNA-seq experiments in each condition were performed in duplicate and triplicate, respectively (Additional file 6: Table S5).

### Gene expression data

Whole-genome gene expression analysis from *Rnf40* ^+/+^ and *Rnf40* ^–/–^ MEFs were generated as previously described [12]. Sequences were mapped to the mouse reference transcriptome (UCSC mm9) and differential gene expression of each sample was normalized using DESeq. Significant differentially expressed genes were classified as follows: downregulated genes (down), baseMean > 15, *p* value < 0.05, log_2_ fold change < –1; upregulated genes (up), baseMean > 15, *p* value < 0.05, log_2_ fold change > 1; unchanged genes (unch), baseMean > 15, *p* value > 0.8, –0.2 < log_2_ fold change < 0.2.

The enrichment scores were calculated using GSEA as described before [59] and gene expression data were sorted by fold changes under *Rnf40* ^–/–^ versus *Rnf40* ^+/+^ conditions. Gene Ontology (GO) enrichment analysis for significantly downregulated and upregulated gene clusters were performed using DAVID 6.7 [60]. The significant enriched GO terms (FDR < 0.05) were shown as a bubble plot generated from REViGO [61].

### ChIP-seq data

The ChIP-seq raw data were mapped to the mouse reference genome (UCSC mm9) using Bowtie (version 1.0.0) [62]. To identify significant peaks, we used Model-based Analysis of ChIP-seq (MACS) (version 1.0.0) for peak calling with the input of each condition as control and *p* value < 0.00001 cutoff for peak detection [63]. Coverage was determined by normalizing the filtered reads per hundred million. The bigwig data were visualized in Integrative Genomics Viewer (version 2.3.14) [64]. The tables containing mouse genome elements (TSS, gene bodies, etc.) and CpG island were obtained from UCSC Table Browser [65]. The average signal of H3K4me3, H3K27me3, and H3K27ac near TSS (±1 kb) and H2Bub1 in gene bodies were computed using ComputeMatrix in deepTools [66]. The heatmapper in deepTools was used to create heatmaps of each ChIP. CEAS (version 1.0.0) and aggregate profile analyses were performed in Galaxy/Cistrome [67]. The H3K27me3 targeted distal regions were obtained by considering only the regions further than 5 kb upstream or downstream of gene bodies. Active enhancers were defined as enriched for (+) H3K4me1 and H3K27ac but negative (–) for H3K4me3 enrichment. Differential binding (DiffBind) analysis of H3K27me3 near TSS (±1 kb) and distal regions or H3K27ac on enhancers under *Rnf40* ^+/+^ versus *Rnf40* ^–/–^ conditions was performed as described before [68]. Enhancer associated coding genes were identified using the Genomic Regions Enrichment of Annotations Tool (GREAT version 3.0.0) [69]. Sequence-based motif analysis for upregulated genes associated with enhancers in *Rnf40* ^–/–^ MEFs was performed using oPOSSUM (version 3.0) [70]. The input file contained the regions (±150 bp) surrounding H3K27ac peak centers on enhancers associated with genes upregulated in *Rnf40* ^–/–^ MEFs. The remaining enhancers not increased following *Rnf40* deletion were utilized as background. Broad H3K4me3 peak regions and peak height were determined using MACS2 [63]. Genes with the top 5% broadest H3K4me3 domains were defined as broad H3K4me3 genes, while genes with the top 5% H3K4me3 occupancy, not overlapping with the broach domain peaks were defined as “sharp” H3K4me3 peaks (see Fig. 2h).

## Additional files

Additional file 1: Figure S1. Loss of Rnf40 significantly alters other histone modifications on genes displaying low or moderate levels of H2Bub1 (Related to Fig. 1). Figure S2 H2Bub1 coordinates H3K4me3 (Related to Fig. 2). Figure S3 H3K4me3 width is linked to transcription elongation rate (Related to Fig. 3). Figure S4 H2Bub1 and broad H3K4me3 domain associate to development-related gene transcription (Related to Fig. 4). Figure S5 RNF40 loss associates to broad activation of PcG repressive targets (Related to Fig. 5). Figure S6 RNF40 loss leads to H3K27me3 decreasing and broad activation of EZH2 targeted genes (Related to Fig. 6). Figure S7 H3K4me3 and H3K27me3 occupancy on Hox genes (Related to Fig. 7). Figure S8 The effect of RNF40 loss on enhancer (Related to Fig. 8). (DOCX 9999 kb)
Additional file 2: Table S1. The gene symbol of selected gene groups in this study including upregulated genes, downregulated genes, H3K27me3 significantly decreased genes, and EZH2 targeted genes. Genes with broad H3K4me3 peaks, sharp H3K4me3 peaks, and random control genes in MEF. (XLSX 70 kb)
Additional file 3: Table S2. The differential alteration of H3K27ac occupancy following RNF40 deletion in the identified enhancers. The value represents log_2_ normalized H3K27ac signal; FDR < 0.05 denotes significantly changed. Fold change calculated by subtracting Conc_*Rnf40* ^+/+^ from Conc_*Rnf40* ^–/–^. (XLS 5537 kb)
Additional file 4: Table S3. The primers used in this study for qRT-PCR and ChIP-qPCR. (XLSX 18 kb)
Additional file 5: Table S4. Antibodies for western blot and ChIP. (XLSX 17 kb)
Additional file 6: Table S5. High throughout sequence data used in this study. (XLSX 17 kb)
