# Supplementary material for: RNF40 regulates gene expression in an epigenetic context-dependent manner
Source: Genome Biol. 2017 Feb 16;18:32. doi: 10.1186/s13059-017-1159-5 (PMC5314486; doi:10.1186/s13059-017-1159-5)

**Supplemental figure legends**

**Additional file 1: Figure S1. Loss of Rnf40 significantly alters other histone modifications on genes displaying low or moderate levels of H2Bub1. Related to figure 1**

(A) Distribution of H2Bub1 ChIP-seq signals on specific genomic regions (top right) compared to the proportion of these regions in the genome (top left). Average H2Bub1 ChIP profile around TSS, TTS, and gene body (bottom panels). (B) The smoothScatter plots show the relationship between H2Bub1 occupancy on the gene body and gene expression in relation to H3K4me3, H3K27ac, as well as H3K27me3 occupancy surrounding the TSS of gene. The correlation coefficient above each smoothScatter plot was calculated using the ‘Pearson’ method. (C) The conditional *Rnf40*^loxP^ locus after integration and removal of the neomycin cassette. (D) The smoothScatter plots compare average H2Bub1 signals on the gene body to the changing occupancy of H3K4me3, H3K27ac, and H3K27me3 near the TSS. (E) Boxplots show the absolute value of log2-fold changes in H3K4me3, H3K27ac, and H3K27me3 occupancy for genes grouped according to H2Bub1 occupancy as “No”, “L”, “M”, and “H”. p-values were calculated by unpaired Wilcoxon-Mann-Whitney-Test.

**Additional file 1: Figure S2. H2Bub1 coordinates H3K4me3. Related to Figure 2**

(A) MA plot show the differential change of H3K4me3 occupancy in respond to H2Bub1 loss. DiffBind analysis was performed to identify the differentially bound sites of H3K4me3. Red points showed the significant changed regions appeared to have an absolute log_2_ fold change of at least 2 (FDR<0.05). (B) Aggregate profiles show the dynamic alteration of H3K4me3 occupancy with H2Bub1 surrounding the all TSS (±3kb) following H2Bub1 deletion. The red profile indicates the average level of H3K4me3 in *Rnf40*^+/+^ subtracts that in *Rnf40*^-/-^ MEFs. (C) Heatmaps show the occupancy of H2Bub1, H3K27me3, and H3K4me3 at CpG enriched and unenriched promoters (surrounding TSS ± 2kb) in *Rnf40*^+/+^ or *Rnf40*^-/-^ MEFs. CpG island regions were obtained from UCSC (mm9). The ratio of observed to expected CpG (obsExp) was used to prepare CpG heatmap. CpG enriched or unenriched promoters were classified based on the obsExp value by using Kmeans method. (D) Correlation between log_2_ fold change in gene expression and log_2_ fold change in H3K4me3 occupancy. Correlation coefficient factor (R) was calculated by pearson’s method. (E) Correlation between gene expression level and H3K4me3 breadth in *Rnf40*^+/+^ MEFs. (F) Cumulative distribution plots of gene proportion for the log_2_ ratio of H3K4me3 width to height (log_2_ width/height) in *Rnf40*^+/+^ and *Rnf40*^-/-^ MEFs. p-value was calculated using unpaired Wilcoxon-Mann-Whitney-Test. (G) Correlation between RNF40-dependent gene expression alteration and their H3K4me3 widespread shortening following H2Bub1 deletion. (H) Aggregate profiles show the average occupancy of H3K4me3 surrounding TSS (±6 kb) at genes with broad, sharp, and random control H3K4me3 in wildtype MEFs. (I – K) Aggregate profiles show the effects of H2Bub1 loss on the occupancy of H3K4me3 in genes with broad H3K4me3, random control, and sharp H3K4me3 in MEFs.

**Additional file 1: Figure S3. H3K4me3 width is linked to transcription elongation rate. Related to Figure 3**

(A) Aggregate profiles show pol II occupancy on genes with broad, random control, and sharp H3K4me3 from TSS to TES in *Rnf40*^+/+^ MEFs. (B and C) Boxplots compare H3K4me3 width (B) or height (C) between genes with top 25% and bottom 25% elongation rate in HeLa cells. The genes with top 25% or bottom 25% elongation rate in HeLa cells were defined before [[1](#_ENREF_1)]. (D) plot profile show the width and height of H3K4me3 peaks near TSS in BMDM cells. Genes with broad, sharp, and random control were defined as Figure 2H. (E - G) Aggregate profiles show the change of H3K4me3 occupancy in genes with broad H3K4me3, random control, and sharp H3K4me3 in respond to Wdr82 deletion in BMDM cells. (H) Aggregate profiles show H2Bub1 occupancy from TSS to TES within genes with broad, random control, and sharp H3K4me3 domain in *Rnf40*^+/+^ MEFs. (K) qRT-PCR analysis of *Myl9* and *Psrc1* genes in *Rnf40*^+/+^ and *Rnf40*^-/-^ MEFs. Expression data was analyzed as Figure 3I. (I and J) ChIP-qPCR for H2Bub1 and H3K4me3 at different regions of *Myl9* and *Psrc1* genes. The regions were pointed out in Figure 3F. Site1 (S1), upstream of TSS; Site2(S2), downstream of TSS; Site3(S3), gene body. Data were analyzed as Figure 3G.

**Additional file 1: Figure S4. H2Bub1 and broad H3K4me3 domain associates to development-related gene transcription. Related to Figure 4**

(A and B) The Bubble plots show GO terms of RNF40-dependent genes and the broadest H3K4me3 occupied genes in MEFs. The GO terms with FDR less than 0.05 are shown here. Bubble color indicates p-value; bubble size indicates the frequency of the GO term in the underlying GO database. Color key of p-value shows at the right side. (C) Venn diagram shows the shared numbers between the broadest H3K4me3 marked genes and upregulated genes (up) or downregulated genes (down). (D) GO terms of RNF40-dependent genes with broad H3K4me3 domain. The GO terms with p-value less than 0.001 are shown here. X axis shows the –log_10_ p-value of each GO term.

**Additional file 1: Figure S5. RNF40 loss associates to broad activation of PcG repressive targets. Related to figure 5**

(A and B) GSEA of mRNA expression data shows a significant enrichment of PRC1-suppressed genes in DAOY cells (A) or PRC2-suppressed genes in TIG3 cells (B) for genes upregulated in *Rnf40*^-/-^ MEFs. NES, normalized enrichment score; FDR, false discovery rate. (C) The scatter plot shows that moderate levels of H2Bub1 on the *Ezh2* gene are associated with RNF40-dependent changes in its expression. The red points denote transcripts encoding the PRC2 subunits EZH1, EZH2, EED, and SUZ12. (D and E) qRT-PCR analysis of *Rnf40* and *Ezh2* in the small intestine, colon, and lung from mouse with or without tamoxifen treatment. Data are represented as mean ± SD (n = 2). *p < 0.05, **p<0.001, unpaired two-tailed t-test. (F) Western blot analysis of whole protein extracts from the small intestine and colon of mice with (ko) or without (con) tamoxifen treatment using antibodies against EZH2, H2Bub1, H3K27me3, and HSC70 (loading control). (G) hMSC transferred with RNF40-specific siRNA were induced to differentiate into adipocytes for 5 days. (H) hMSC treated with 5 µM of CDK9 inhibitor (LDC000067) were induced to differentiate into adipocytes for 5 days. EZH2 expression was analyzed by qRT-PCR. Data show mean ± SD, n = 3; *p<0.05, **p<0.001, unpaired two-tailed t-test. (I) GSEA of RNA-seq data for adipocyte differentiation transferred with siRNA targeting RNF40 or non-targeting siRNA [[2](#_ENREF_2)]. The table shows the top 20 enriched gene sets. The marked gene sets are polycomb-suppressed genes. (J – L) MEFs were cultured in DMEM supplemented with 10% FBS (high serum) or 0.5% FBS (low serum) for 24 hours. In order to induce *Rnf40* deletion, cells were cultured with 250 nM of 4-OHT in DMEM supplemented with low and high serum for another 48 hours. The expression of *Rnf40*, *Ccne1*, and *Ezh2* were analyzed by qRT-PCR. Data were further showed as G and H.

**Additional file 1: Figure S6. RNF40 loss leads to H3K27me3 decreasing and broad activation of EZH2 targeted genes. Related to Figure 6**

(A and B) Boxplots compares H3K27me3 level surrounding TSSs (±1kb) (A) and distal regions (B). The level of H3K27me3 on the given regions was calculated via DiffBind analysis. The size of each boxplot indicates the numbers of genes. (C) The heatmaps show EZH2 and H3K27me3 level surrounding TSS (±5kb) of EZH2 targeted genes. Genes are sorted according to EZH2 level in descending order. Color key of the heatmaps is shown at their right side. (D) Heatmap shows the expression of genes displaying significantly decreased H3K27me3 near the TSS. The given values are log2-fold change of gene expression (*Rnf40*^-/-^ / *Rnf40*^+/+^). (E) GSEA of mRNA expression data shows a significant enrichment of EZH2-targeted genes for genes upregulated in *Rnf40*^-/-^ MEFs. (F and G) qRT-PCR analysis of *Tgfa*, *Kcnc3*, *Chd5*, and *Psrc1* in wildtype, *Rnf40*-deleted and EZH2i MEFs. Data show mean ± SD, n = 3; *p<0.05, **p<0.001, unpaired two-tailed t-test. (H) qRT-PCR analysis of *Nat8l* expression in colon from mouse with (*Rnf40*^-/-)^ or without (*Rnf40*^+/+^) tamoxifen treatment. Data show mean ± SD, n = 2; **p<0.001, unpaired two-tailed t-test. (I) The ChIP-seq profiles for H2Bub1, H3K4me3, H3K27me3, H3K27ac, SUZ12, and EZH2 on the *Chd5*, *Kcnc3* and *Tgfa* genes. (J) The Bubble plots show GO terms of RNF40-supressed genes in MEFs. The GO terms with FDR less than 0.05 are shown here.

**Additional file 1: Figure S7. H3K4me3 and H3K27me3 occupancy on Hox genes. Related to figure 7**

(A and B) ChIP-qPCR analysis for H3K4me3 and H3K27me3 occupancy at promoters of *Hoxc13* and *Hoxc6* in wildtype, *Rnf40*-deleted, and EZH2i MEFs. Data were analyzed as Figure 7C. (C) ChIP-seq profiles show H3K27me3 and H3K4me3 occupancy on *HOXB2* and *HOXC10* in undifferentiated hMSC (un diff) and differentiated into adipocytes (ADI diff).

**Additional file 1: Figure S8. The effect of RNF40 loss on enhancer. Related to figure 8**

(A) Venn diagram show the shared numbers of upregulated (upre.), downregulated (downer.), H3K4me3 enriched (H3K4me3+), and H3K27me3 enriched (H3K27me3+) genes. (B and C) Venn diagrams identified the numbers of active enhancers displaying enriched (+) H3K4me1 and H3K27ac but unenriched (-) H3K4me3 in *Rnf40*^+/+^ or *Rnf40*^-/-^ MEFs. Venn diagram shows the numbers shared by H3K27ac and H3K4me3 (B). Venn diagram shows the numbers shared by H3K4me3 negative & H3K27ac positive regions (H3K4me3^-^/H3K27ac^+^) and H3K4me1 regions (C). (D) Boxplots show the alteration of H3K27ac average signal on enhancers following RNF40 deletion. The average signal of H3K27ac on each enhancer was calculated via DiffBind analysis. (E) The heatmaps show H3K4me1, H3K27ac and H3K27me3 level on RNF40-suppressed gene associated enhancers. The center of each heatmap denotes the centers of H3K4me1 peaks. Regions are sorted according to H3K4me1 level in descending order. Color key of the heatmaps is shown at their right side. (F) Venn diagram shows the shared genes between “down” Gene cluster and RNF40-dependent enhancer associated genes (displaying a decreasing H3K27ac occupancy following RNF40 deletion) (G) Venn diagram shows the shared regions between enhancer in MEFs and FOXL2 binding sites in mouse granulosa cells. (H) Venn diagram shows the shared genes between RNF40-suppressed enhancer associated and FOXL2 enriched enhancers associated upregulated genes. (I) qRT-PCR analysis of *Rnf40*. *Ezh2*, *Esr2*, and *Efna5* in mouse ovary. Each group only included one mice. (J) The ChIP-seq profiles for H2Bub1, H3K4me3, H3K27me3, H3K27ac, H3K4me1 and FOXL2 on *Esr2* gene.

**Supplemental Reference**

1. Fuchs G, Voichek Y, Benjamin S, Gilad S, Amit I, Oren M: **4sUDRB-seq: measuring genomewide transcriptional elongation rates and initiation frequencies within cells.** *Genome Biol* 2014, **15:**R69.

2. Karpiuk O, Najafova Z, Kramer F, Hennion M, Galonska C, Konig A, Snaidero N, Vogel T, Shchebet A, Begus-Nahrmann Y, et al: **The histone H2B monoubiquitination regulatory pathway is required for differentiation of multipotent stem cells.** *Mol Cell* 2012, **46:**705-713.


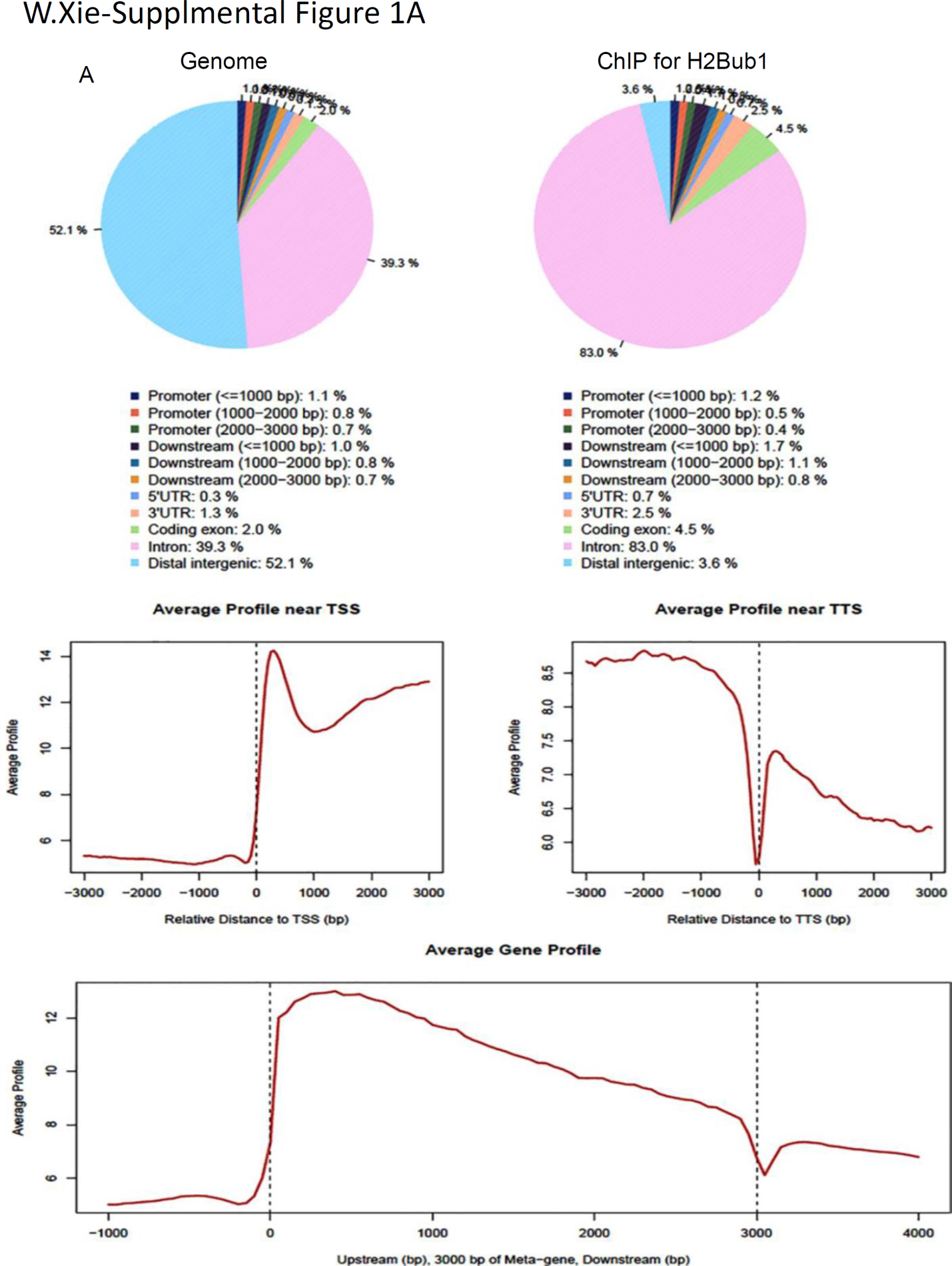


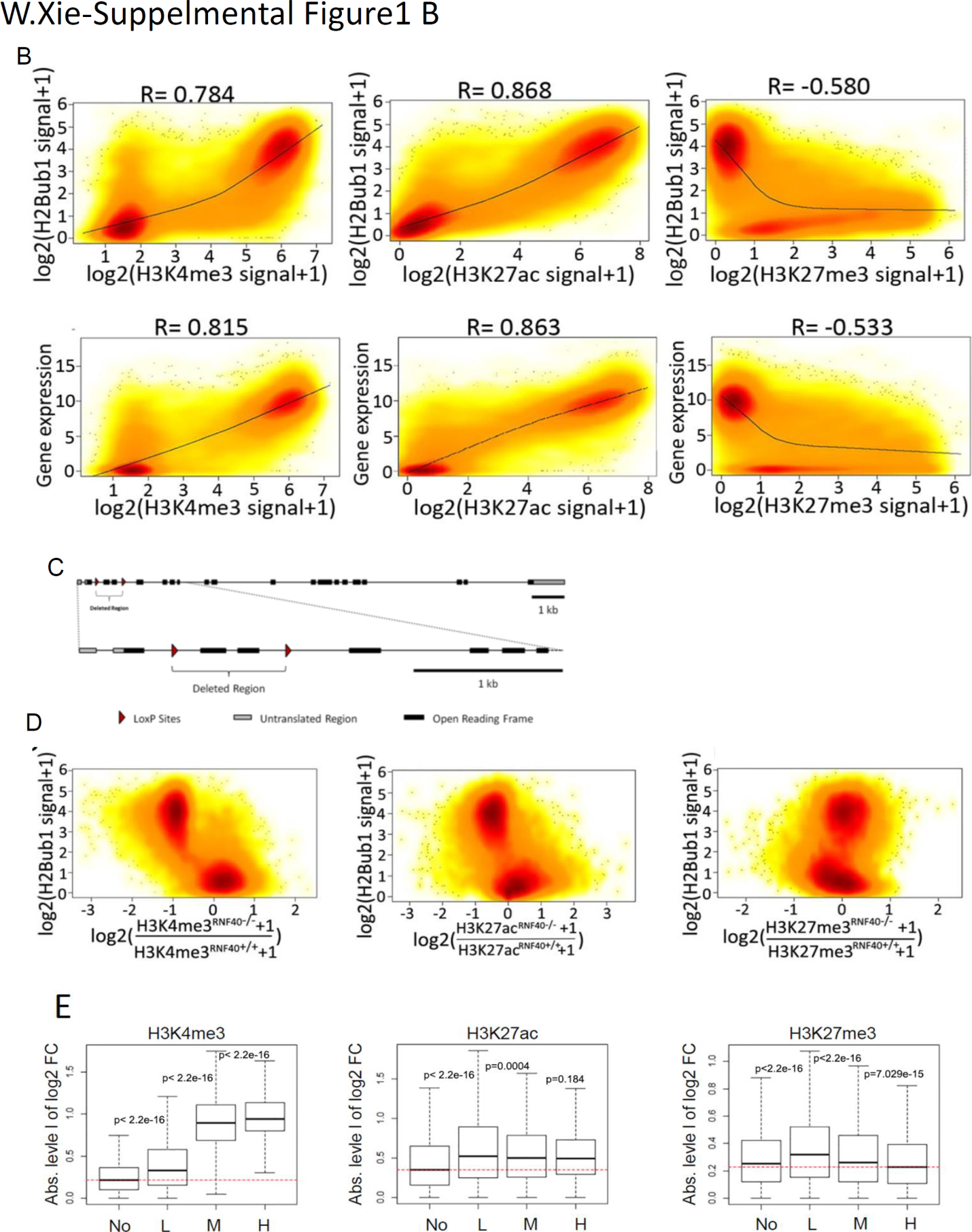


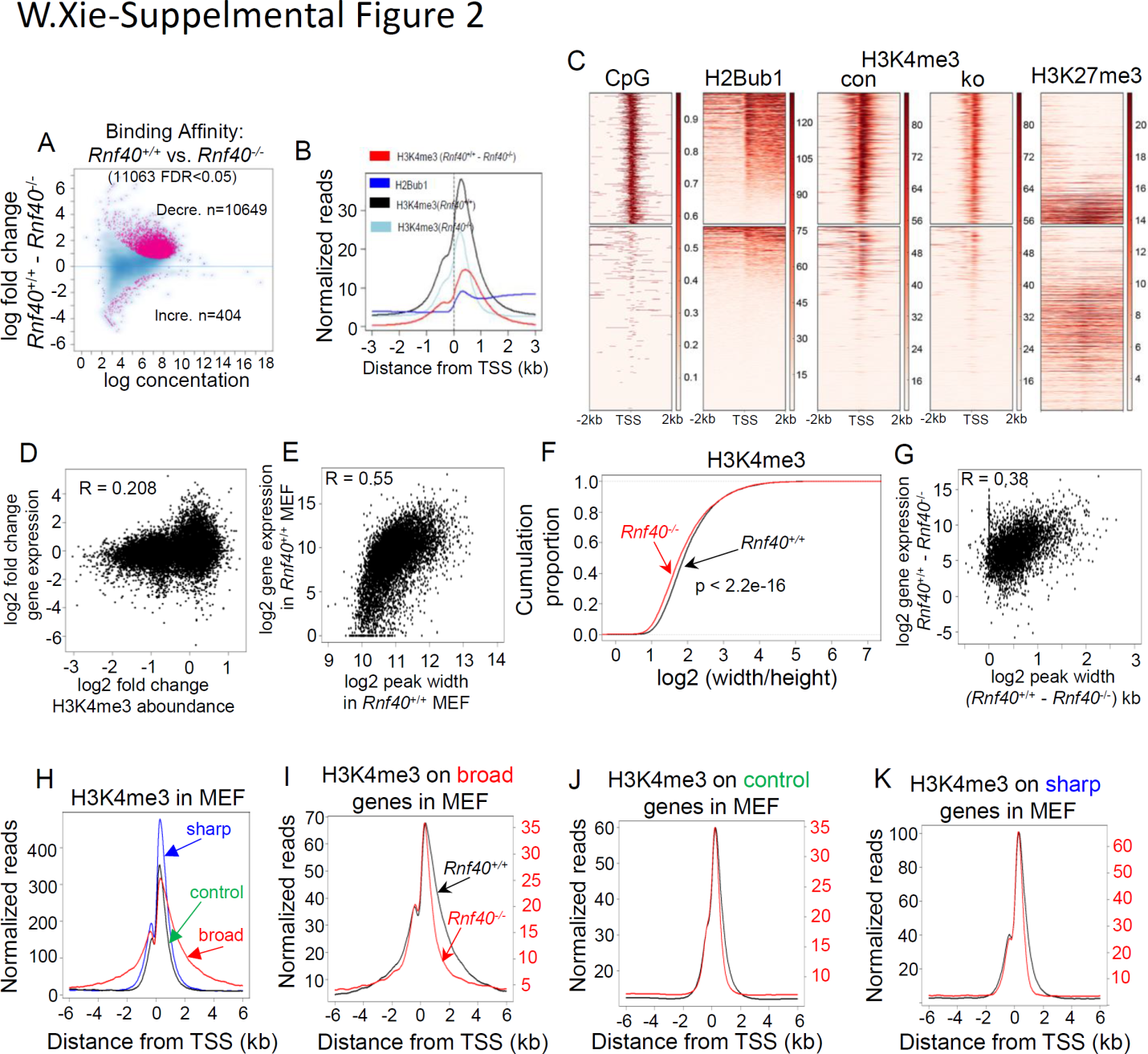


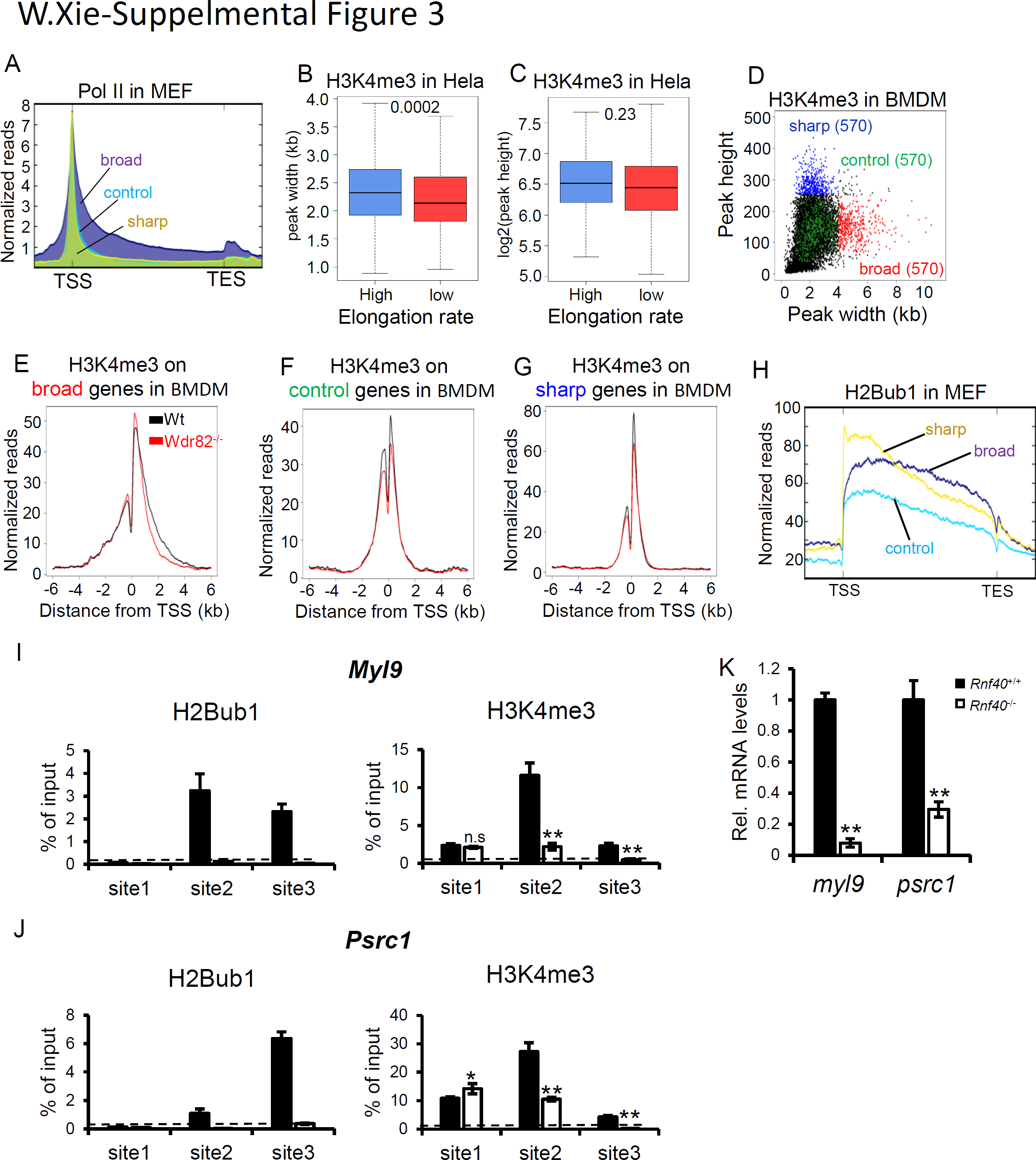


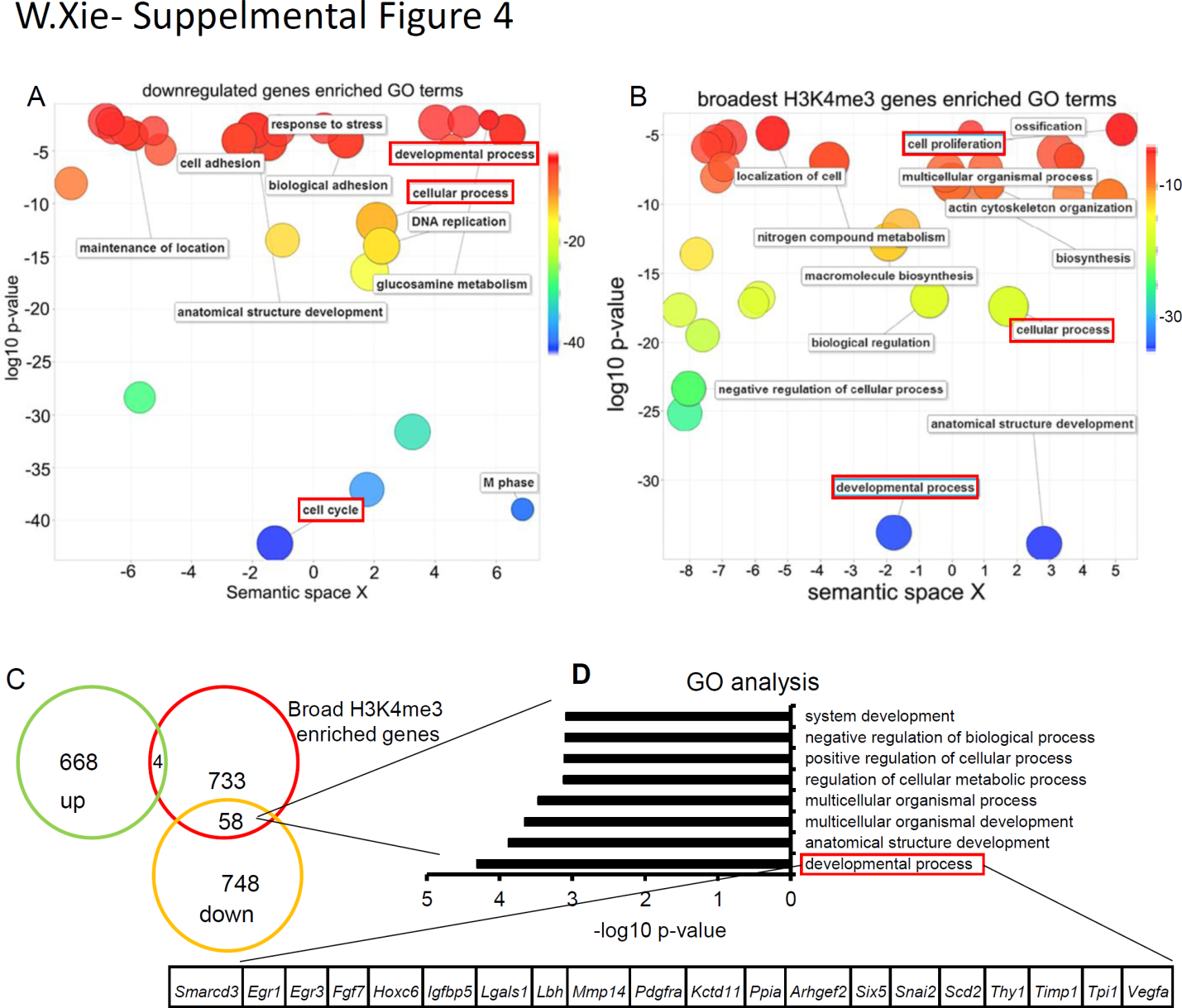


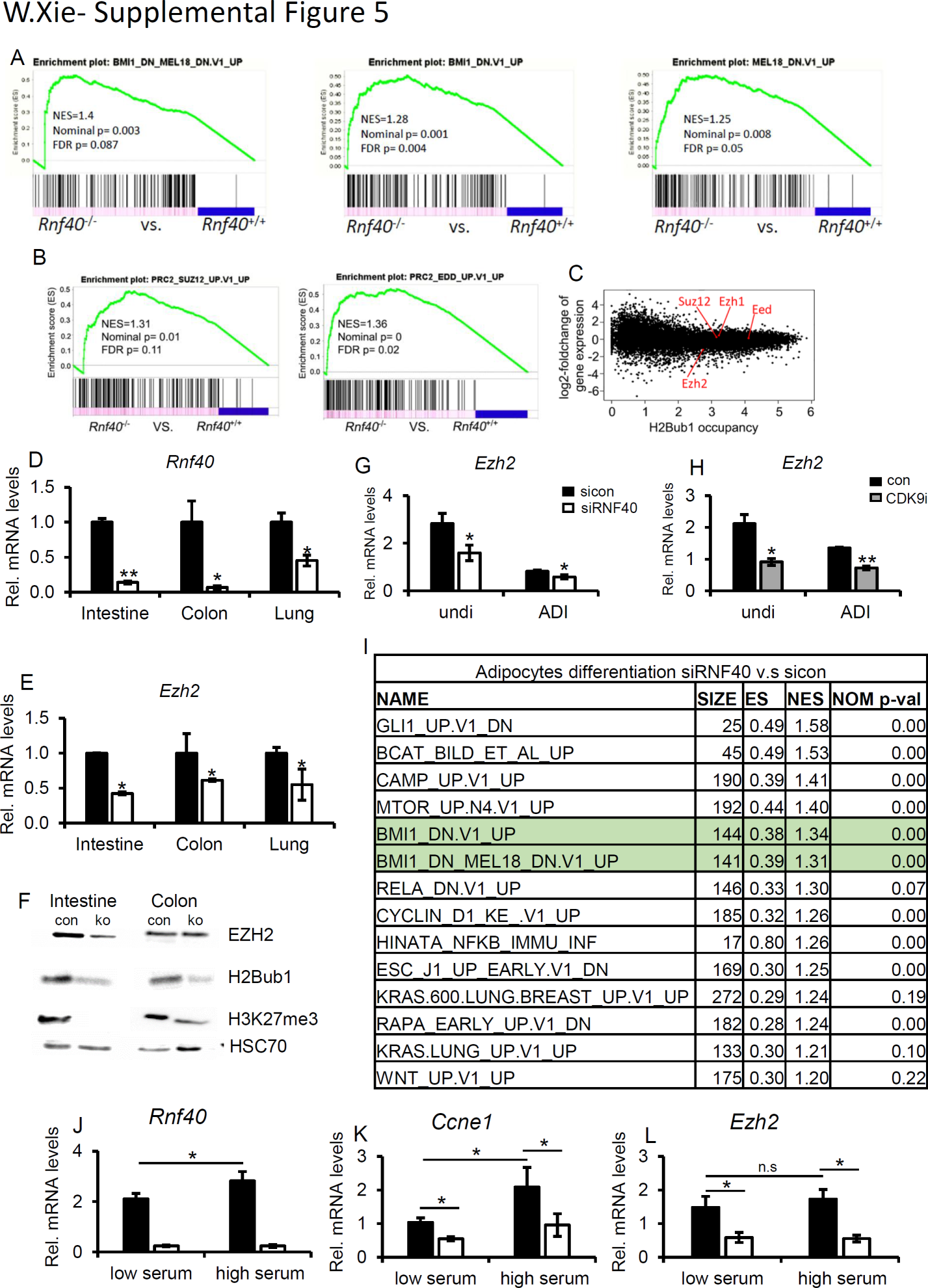


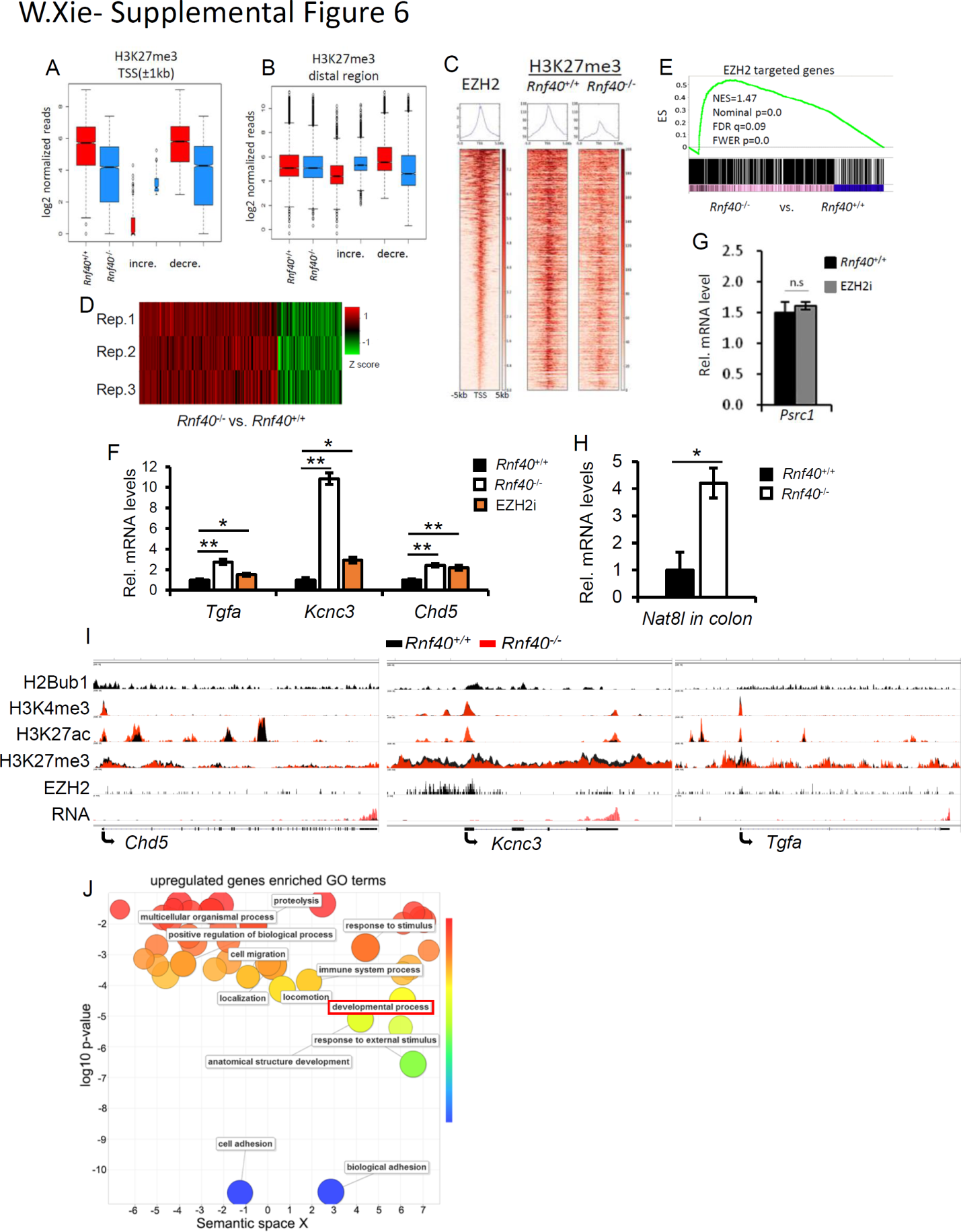


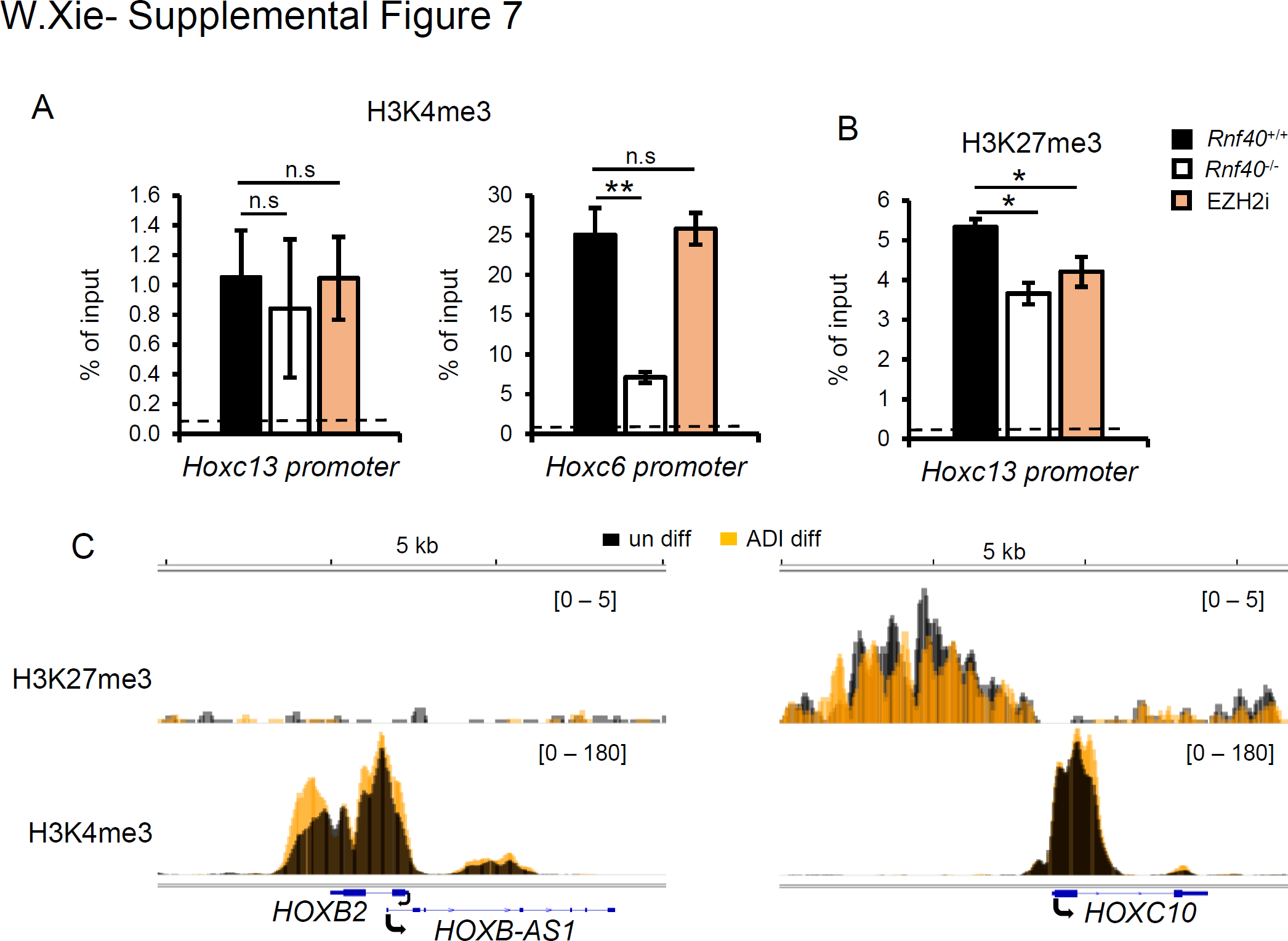


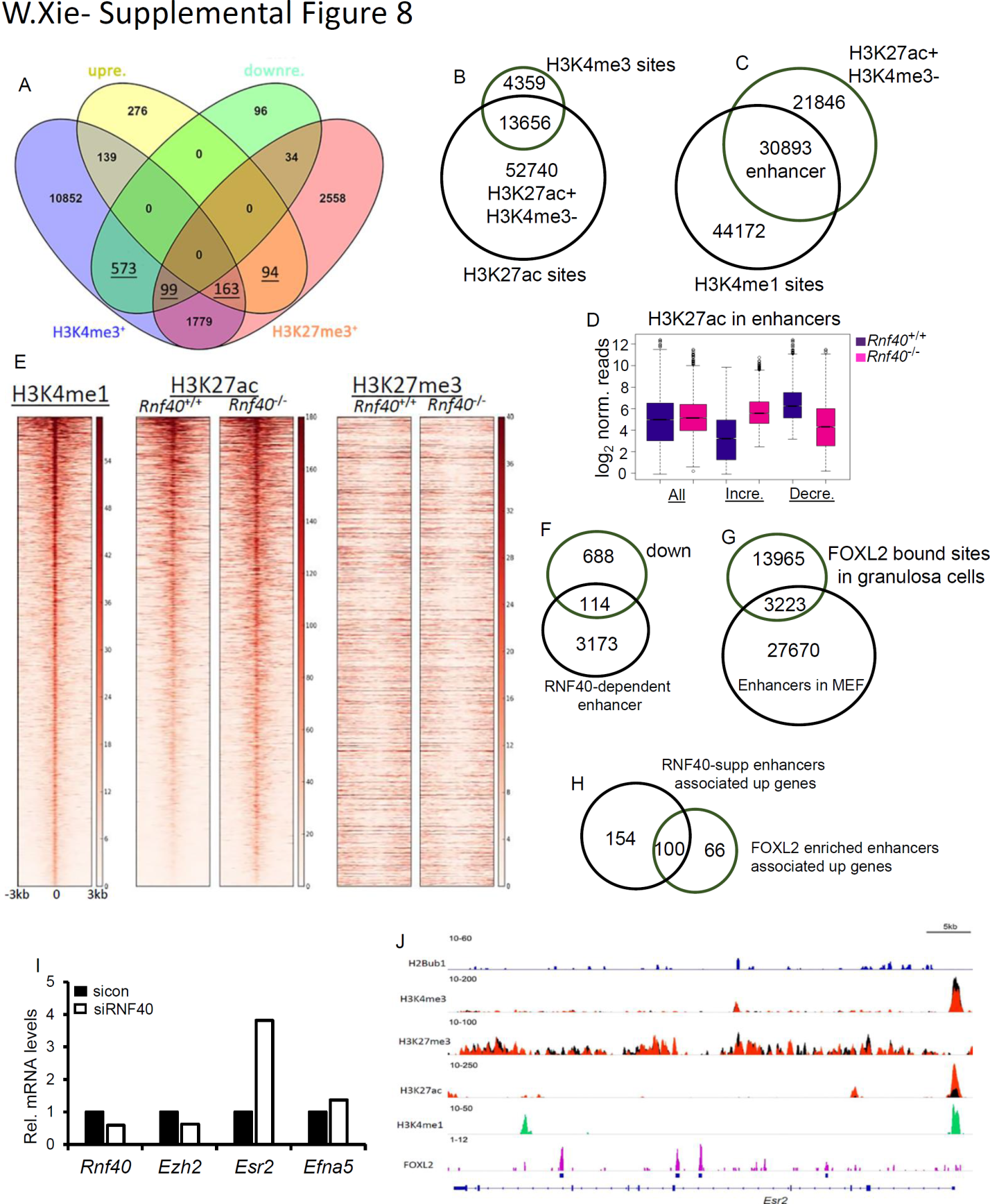

Supplement: Additional file 1: Figure S1. — Loss of Rnf40 significantly alters other histone modifications on genes displaying low or moderate levels of H2Bub1 (Related to Fig. 1). Figure S2 H2Bub1 coordinates H3K4me3 (Related to Fig. 2). Figure S3 H3K4me3 width is linked to transcription elongation rate (Related to Fig. 3). Figure S4 H2Bub1 and broad H3K4me3 domain associate to development-related gene transcription (Related to Fig. 4). Figure S5 RNF40 loss associates to broad activation of PcG repressive targets (Related to Fig. 5). Figure S6 RNF40 loss leads to H3K27me3 decreasing and broad activation of EZH2 targeted genes (Related to Fig. 6). Figure S7 H3K4me3 and H3K27me3 occupancy on Hox genes (Related to Fig. 7). Figure S8 The effect of RNF40 loss on enhancer (Related to Fig. 8). (DOCX 9999 kb) [file 13059_2017_1159_MOESM1_ESM.docx]
